# *Helicase-like transcription factor (HLTF)*-deleted CDX/TME model of colorectal cancer increased transcription of oxidative phosphorylation genes and diverted glycolysis to boost S-glutathionylation in lymphatic intravascular metastatic niches

**DOI:** 10.1371/journal.pone.0291023

**Published:** 2023-09-08

**Authors:** Dalia Martinez-Marin, Rebecca A. Helmer, Gurvinder Kaur, Rachel L. Washburn, Raul Martinez-Zaguilan, Souad R. Sennone, Jannette M. Dufour, Beverly S. Chilton

**Affiliations:** 1 Department of Cell Biology and Biochemistry, Texas Tech University Health Sciences Center, Lubbock, Texas, United States of America; 2 Department of Immunology and Molecular Microbiology, Texas Tech University-Health Sciences Center, Lubbock, Texas, United States of America; 3 Current address: Garrison Independent School District, Garrison, Texas, United States of America; 4 Department of Medical Education, Texas Tech University Health Sciences Center, Lubbock, Texas, United States of America; 5 Department of Cell Physiology and Molecular Biophysics, Texas Tech University Health Sciences Center, Lubbock, Texas, United States of America; 6 Texas Center for Comparative Cancer Research, Texas Tech University School of Veterinary Medicine, Amarillo, Texas, United States of America; 7 School of Medicine Cancer Center, Texas Tech University Health Sciences Center, Lubbock, Texas, United States of America; Columbia University Irving Medical Center, UNITED STATES

## Abstract

*Helicase-like transcription factor* (*HLTF*) also known as *SMARCA3*, protects genome integrity. A tumor suppressor, *HLTF* is expressed in tumor cells but not in the tumor microenvironment (TME) in early-stage colorectal cancer (CRC). With disease progression, there is high concordance between epigenetic silencing of *HLTF* in CRC cells and negligible *HLTF* expression in the TME. We developed a cell line-derived xenograft (CDX) model and show for the first time that *HLTF*-deletion in cancer cells and the TME results in metabolic reprogramming that mitigates oxidative stress in lymphatic intravascular metastatic niches. The two metabolic pathways that derive energy from glucose—glycolysis and oxidative phosphorylation (OXPHOS)—are variously utilized by cancer cells depending upon the TME. *HIF-1α*, a master regulator of glycolysis, was eliminated from a role in reprogramming metabolism to satisfy CDX energetic requirements by RNAseq and spatial transcriptomics. Variability in the gut microbiome, with a putative role in altered metabolism, was also eliminated. *HLTF*-deleted cancer cells recovered from DNA damage at a transcriptomic level induction of DNA repair and OXPHOS genes linked to an amoeboid-associated phenotype at the tumor border (confocal microscopy). *HLTF*-deleted cancer and endothelial cells of lymphatic (PDPN) intravascular niches in the TME shared a site-specific protein S-glutathionylation signature (2D DIGE, MALDI-TOF/TOF mass spectrometry) for three glycolytic enzymes (PGK1 Cys^379/380^, PGAM1 Cys^55^, ENOA1 Cys^119^) that diverted glycolysis in support of continued glutathione biosynthesis. The collective absence of *HLTF/Hltf* from tumor and TME achieved redox homeostasis throughout the CDX and promoted metastasis.

## Introduction

Colorectal cancer (CRC) is the third most common cause of cancer-related deaths worldwide [1]. The preference for early detection and surgical removal of CRC emphasizes the challenges of managing metastatic disease with its underlying risk of therapeutic resistance [2]. Of patients that present with CRC, ~20% already have metastatic disease, and an additional 40% with local disease will later develop metastases. Patients with metastatic CRC have a dismal 5-year survival rate < 20%.

The fact that aberrant DNA methylation in CRC cells increased cell-free serum DNA levels [3] drove the search for novel methylation biomarkers with therapeutic potential. *HLTF* emerged as a promising candidate because it is epigenetically silenced in 43% of CRC cells [4] but not in normal cells. Hypermethylated *HLTF* DNA in serum samples significantly correlated with tumor size, more aggressive tumors, advanced stage (III or IV) metastatic disease, including micro-metastasis, and shorter survival [5, 6]. The shift in the global focus from ‘only’ tumor cells to mechanisms shared by tumor cells and cells of the tumor microenvironment (TME) showed negligible HLTF protein in fibroblasts and endothelial cells of the TME. Reference component RNAseq analysis of single-cell transcriptomes from eleven primary tumors and matched normal mucosa identified fibroblast populations—normal, myofibroblasts, and cancer-associated fibroblasts—based on gene expression profiles [7]. *HLTF* was not detected in any of the fibroblast populations despite measured changes in mRNA levels between normal and tumor cells. HLTF in normal endothelial and lymphatic endothelial cells (Human Protein Atlas), is purported to have a scaffolding function with LINC00607, an endothelial-specific long non-coding RNA, in the regulation of gene transcription [8]. However, *HLTF* is not expressed in endothelial cells in CRC [9].

Because the effects of *HLTF*-deletion from tumor cells and cells of the TME has never been studied *in vivo*, we developed a HCT116 cell line-derived xenograft model (CDX) of tumorigenesis. In the first study [10], immunodeficient (ID) *Hltf*-deleted and control male mice received direct orthotopic cell microinjection (OCMI) of *HLTF*^+/+^HCT116-Red-Fluc cells in the cecum. RNAseq with species-specific mapping unambiguously differentiated gene expression in tumor cells from cells of the TME and showed *Hltf*-deletion from the mouse TME reprogramed the *HLTF*^+/+^ human tumor transcriptome and initiated a gene deletion-specific program for metastasis.

Whole transcriptomic sequencing provided an exceptional view of the dynamic interaction between cells of the tumor and TME. However, gene information in a spatial context was lost due to tissue homogenization. Spatial transcriptomics on formalin-fixed paraffin embedded (FFPE) tissue captures positional gene patterns within intact tissue. In this second study, we utilized high-throughput RNAseq with species-specific mapping in conjunction with spatial transcriptomics in a direct comparison of early-stage vs late-stage primary tumors, i.e., *HLTF*^+/+^CDX vs *HLTF*^-/-^CDX in ID *Hltf*-deleted mice. First, we generated an isogenic *HLTF*^*-/-*^HCT116-Red-FLuc cell line that was otherwise identical to the parental line. Next, we established primary *HLTF*^-/-^CDX tumors in ID *Hltf*-deleted male *Rag2*^*-/-*^*IL2rg*^*-/-*^ mice by direct OCMI of the cells into the cecum. Combinatorial use of RNAseq with species-specific mapping, spatial transcriptomics, immunohistochemistry and 2D DIGE, MALDI-TOF/TOF mass spectrometry provided a holistic understanding of a major mechanism coordinating escape from oxidative stress in lymphatic intravascular metastatic niches when *HLTF* expression is silenced in tumor cells and cells of the TME.

## Materials and methods

### Reagents and kits

Microbiome collection kits were obtained from TransnetYX (Cordova, TN). McCoy’s 5a Medium (30–2007) and fetal bovine serum (30–2020) were purchased from American Type Culture Collection (ATCC, Manassas, Virginia). Puromycin (ant-pr) was purchased from InvivoGen (San Diego, CA). XenoLight D-luciferin potassium salt (122799) was purchased from PerkinElmer (Waltham, MA). ThermoScientific (Waltham, MA) was the source of glutathione mouse monoclonal antibody (D8; MA1-7620) and the Pierce™ reagent HENS buffer (90106). PerkinElmer (Waltham, MA) was the source of Bioware® Brite Cell Line HCT116 Red-FLuc (BW124318). 10X Genomics (Pleasanton, CA) was the source of all Visium reagent kits for whole transcriptome profiling of intact formalin fixed paraffin embedded (FFPE) tissue sections including test slide (PN-1000347), slide kit (PN-1000188), reagent kit (PN-1000361), human transcriptome probe kit (PN-1000361), accessory kit (PN-1000194) and dual index kit TS Set A (PN-1000251). KAPA SYBR FAST qPCR master mix (KK4600) was purchased from Roche Diagnostic Corporation (Indianapolis, IN). SPRIselect (B23317) was purchased from Beckman Coulter Life Sciences (Indianapolis, IN). ProLong Gold antifade reagent with DAPI (P36935) and Streptavidin-FITC (19538–050) were from Invitrogen by ThermoFisher Scientific. 3-(N-Maleimidylpropionyl) biocytin (MPB) was from Santa Cruz Biotechnology (sc-216373). The following were purchased from Millipore Sigma: NADPH, tetrasodium salt (481973), Glutaredoxin-S2, *E*. *coli* (GRX1, 354406), Glutathione reductase (GSSG, G3664), and glutathione (GSH, Y0000517). Primary and secondary antibodies used with FFPE tissue sections in immunohistochemistry (IHC-P) and confocal immunocytochemistry (ICC/IF) are listed in Table 1. All protocols are accessible in protocols.io (doi.org/10.17504/protocols.io.ewov1oe37lr2/v1).

**Table 1:** Source, application and concentration of antibodies.

Primary Antibodies	Secondary Antibodies
Rabbit polyclonal HLTF antibody (NBP1-83256) Novus Biologicals (1:100) Rabbit polyclonal CYTB (55090-1-AP) ThermoFisher Scientific (1:50) Rabbit polyclonal PGAM1 (16126-1-AP) ThermoFisher Scientific (1:50) Rabbit polyclonal ANXA1 (71–3400) ThermoFisher Scientific (1:20) Rabbit monoclonal γH2AX-phospho S139 (ab81299) Abcam (1:50) Rabbit monoclonal ENO1 (ab227978) Abcam (1:2,000) Rabbit recombinant monoclonal mouse-specific PDPN (MA5-29742) ThermoFisher Scientific (1:500)	IHC-P: Vector Laboratories RTU Biotinylated Goat Anti-rabbit IgG (H+L) (BP-9100-50)
Mouse monoclonal PGAM1 (67470-1-1g) ThermoFisher Scientific (1:200)	ICC/IF: Goat anti-mouse, Alexa Fluor™ Plus 647 (A-32728) ThermoFisher Scientific (~4 μg/mL)
Rabbit polyclonal ANXA1 (71–3400) ThermoFisher Scientific (1 μg/mL)	ICC/IF: Donkey anti-rabbit, Alexa Fluor™ 555 (A-31572) ThermoFisher Scientific (~4 μg/mL)

### Gut microbiome

Individual mice were allowed to defecate normally in autoclaved cages with no bedding. Using a sterile 26Gx1/2 needle (one per mouse), the first two fecal pellets per mouse were submerged in stabilization buffer in a barcoded sample collection tube. Collection tubes were shipped to TransnetYX for DNA extraction (Qiagen DNeasy 96 PowerSoil Pro QIAcube HT kit/protocol), library preparation (KAPA HyperPlus protocol), and sequencing. High molecular weight genomic DNA captured the true microbial diversity of stool samples from two cohorts: six- to eight-week-old *Hltf*^*+/+*^ (n = 9) and *Hltf*^*-/-*^ (n = 5) immune deficient (ID) male mice. Libraries were sequenced using Illumina NovaSeq at a dept of 2 million read pairs (2x150 configuration) sufficient for species and strain level taxonomic resolution. FASTQ files were uploaded onto One Codex analysis software [11] and analyzed against the One Codex database of ~127K whole microbial reference genomes. Classification results were filtered through several statistical post-processing steps to eliminate false positive results caused by contamination or sequencing artifacts. Mouse 12K metagenome-assembled genomes were included to screen out host reads. One Codex annotated metagenomes and generated a taxa plot of the top 10 species for all samples, and calculated alpha (within-sample) family diversity for comparison of the two cohorts.

### *HLTF*-deleted isogenic HCT116-Red-FLuc cells

Bioware® Brite Cell Line HCT116-Red-FLuc is a luciferase-expressing derivative of a highly metastatic cell line (HCT116 parental line, CCL-247). PerkinElmer’s recommendation that the Bioware® Brite Cell Line HCT116-Red-FLuc only be passaged 10 times, precluded the use of CRISPER/Cas9 to generate a stable knockout of *HLTF*. Thus, System Biosciences (Palo Alto, CA) produced a custom stable cell line with shRNA sequence targeting of *HLTF* [12]. Briefly, synthesized *HLTF* shRNA sequence was cloned into pGreenPuro shRNA expression lenti vector (SI505A-1). HCT116-Red-Fluc cells infected with 10 MOI of *HLTF*-shRNA-SI505 virus (lot#171009–008) yielded puromycin-resistant polyclonal HCT116-Red-FLuc *HLTF*^-/-^ cells. For each CDX experiment, cell stocks in liquid nitrogen at passage 2 were thawed using T25 flasks, expanded in T150 flasks for 2 days, passaged overnight and harvested at 70–75% confluency. We used this protocol as a unified framework for comparative gene expression analysis.

### *Hltf*-deleted mice

Immune competent (IC) global *Hltf* knockout (KO) mice were developed in collaboration with genOway (Lyon, France) as previously described [13]. Speed congenics accelerated the generation of IC *Hltf* KO C57BL/6J mice [14] that were bred (IACUC# 02007) into the recombinase activating gene 2 (Rag2)/common gamma (IL2rg) double knockout background [15] to generate ID *Hltf* KO mice >99% C57BL/6 background genome. ID *Hltf* KO mice were housed in a barrier facility with a 12:12 light/dark cycle, access to food and water *ad libitum* and bedding changes 2–3 times/week. Routine testing of sentinel mice ensured the colony was disease free. All studies and the anticipated mortality were conducted in accord with the NIH Guidelines for the Care and Use of Laboratory Animals, as reviewed and approved by the Institutional Animal Care and Use Committee at Texas Tech University Health Sciences Center (NIH Assurance of Compliance A3056-01; USDA Certification 74-R-0050, Customer 1481, S1 Checklist). TTUHSC’s IACUC (# 02009) specifically approved this study.

For the orthotopic *HLTF*^*-/-*^HCT116 xenograft model, six- to eight-week old ID *Hltf* KO mice (n = 7) received direct OCMI of *HLTF*^*-/-*^HCT116-Red-Fluc cells (2x10^6^ cells/10 μl) between the mucosa and the muscularis layers of the cecal wall. Each surgery was performed with isoflurane (isothesia) and the SomnoSuite® Low-Flow anesthesia system (Kent Scientific) with far infrared warming pads during surgery and recovery. Efforts to minimize suffering included an IP injection of Buprenorphine (Buprenex, 0.1 mg/kg) prior to surgery to manage incisional pain followed by a second dose 4–8 hours later, if needed. The cecum was exteriorized via small midline incision on the linea alba between the rectus abdominus muscles to eliminate bleeding. Non-invasive bioluminescence imaging (BLI) with an IVIS Spectrum *In Vivo* Imaging System was used to validate the quality and accuracy of the injection, and to track and quantify tumor growth and metastasis. Histopathology at necropsy confirmed placement of the inoculum. Mouse behavior and well-being were monitored daily. Tumor growth/metastasis was monitored weekly with BLI.

Mice were placed under continuous isothesia for imaging and killed immediately thereafter (<15 seconds) by cervical dislocation at the humane endpoint of 28 days post CDX establishment. Primary *HLTF*^*-/-*^CDX (hereafter *HLTF*^*-/-*^CDX) tumors were quickly removed, rinsed in physiological saline and either flash frozen for biochemical evaluation (RNAseq, Western blotting, MALDI-TOF/TOF MS, nanoLC-MS/MS) or formalin fixed paraffin embedded (FFPE) for routine histopathology, IHC-P, laser scanning confocal ICC-IF and spatial transcriptomics.

### Microscopy

FFPE *HLTF*^*-/-*^CDX were serially sectioned (3–4 μm) for histopathology, IHC-IP and ICC-IF. Two sections were placed on each slide and deparaffinized prior to staining. Beginning with the first slide, sections on every fifth slide were stained with hematoxylin and eosin (H&E) for evaluation by light microscopy. Sections on alternate slides were processed for IHC-IP with heat-induced epitope retrieval (HIER). Two tissue sections per slide facilitated the use of one section for positive immunostaining, and the companion section for negative (minus primary antibody) control staining. Pairing of primary antibodies with secondary antibodies is shown in Table 1. Nuclei were counterstained (blue) with hematoxylin. ICC-IF (Table 1) was performed with serial sections from the above described FFPE tumors with HIER, aldehyde quench (50 mM NH_4_Cl in PBS), and ProLong Gold DAPI (DNA-specific blue-violet fluorescent dye).

Protein S-glutathionylation (Pr-SSG) was detected *in situ* in FFPE *HLTF*^*-/-*^CDX sections (3–4 μm) with a biotin switch assay [16]. Free thiol groups were blocked in HENS buffer (100mM HEPES, pH 7.8, 1 mM EDTA, 0.1 mM Neocuproine, 1% SDS) containing 20 mM MMTS (methyl methanoethiosulfonate) and 1% triton (vol/vol) for 10 minutes. After three rinses in PBS, S-glutathionylated cysteine groups were reduced by incubation with 27 μg/ml E coli GRX1, 4 U/ml GSSX, 1 mM GSH, 1 mM NADPH in 1 mm EDTA/10mM Tris pH 8 for 20 minutes. After three rinses in PBS, newly reduced cysteine residues were labeled with 1 mM MPB for 60 minutes. Excess MPB was removed with six rinses in PBS and tissue samples were incubated with 0.5 μg/ml streptavidin-FITC for 60 minutes. Excess streptavidin-FITC was removed with 6 rinses in PSB and cover slipped with ProLong Gold antifade reagent with DAPI to stain nuclei. Slides were allowed to cure overnight at room temperature and then stored at -20C until confocal imaging. Controls were treated the same except E. coli GRX1 was omitted from the deglutathionylation step.

### Tumor transcriptome (RNAseq)

*HLTF*^*-/-*^CDX (1 per ID *Hltf* KO mouse x 3 biological replicates) were flash frozen and sent to Genewiz, the next generation sequencing (NGS) division of Azenta Life Sciences (South Plainfield, NJ) RNA-sequencing. Briefly, total RNA was isolated, and evaluated for its integrity and purity with an Agilent Bioanalyzer (Table 2). RNA was polyA selected prior to library preparation (Illumina Stranded mRNA prep) followed by Illumina HiSeq, 2x150 configuration, single index per lane with 350 million raw paired-end (PE) reads per lane, i.e. 40–60 million PE reads per sample (>80% of bases >Q30). Sequence reads were trimmed to remove possible adapter sequences and nucleotides with poor quality using Trimmomatic v.0.36. The trimmed reads were mapped to either the Homo sapiens GRCh38 reference genome or the Mus musculus GRCm38 reference genome on ENSEMBL using the STAR aligner (v.2.5.2b) that detects splice junctions and incorporates them to help align the entire read sequences. BAM files were generated as a result of this step.

**Table 2:** Sample quality control and RNAseq outcome.

Sample ID	OD260/280	RIN^a^	DV200^b^	Yield (Mbases)	Total Reads	%bases >30^c^
ID *Hltf* KO	2.16	9.1	85.62	24,189	80,629,398	91.70
ID *Hltf* KO	2.10	8.6	63.46	25,733	85,775,084	91.24
ID *Hltf* KO	1.50	8.4	88.15	23,777	79,255,475	91.34

^a^RNA integrity number (RIN) from Agilent Bioanalyzer

^b^Percentage RNA fragments >200 nucleotides (Tapestation)

^c^Base calling software assigns quality score (Q) to each base; Q30 = accuracy of the base call is 99.9%

Unique gene hit counts were calculated by using featureCounts from the Subread package v.1.5.2. The hit counts were summarized and reported using the gene_id feature in the annotation file. Only unique reads that fell within exon regions were counted. After extraction, gene hit counts were used for downstream differential gene expression analysis (DESeq2) of *HLTF*^*-/-*^ and *HLTF*^*+/+*^CDX from ID *Hltf* KO mice. Log2 fold changes (L2 FC) were calculated. P-values were obtained with the Wald test, and adjusted p-values (padj) were calculated with the Benjamini-Hochberg method. Genes with padj < 0.05 and absolute L2 FC >1 were called as differentially expressed genes for each comparison. New RNAseq data for *Hltf*^-/-^CDX in ID *Hltf* KO are accessible through NCBI’s Gene Expression Omnibus (GEO) SuperSeries accession number GSE234728, SubSeries GSE234725 (RNA-Seq). RNAseq data for *Hltf*^+/+^CDX in ID *Hltf* KO are accessible through GSE161961.

### Spatial transcriptomics

#### Work flow

five basic steps were necessary to implement spatial transcriptomics technology to quantify inter and intra tumor heterogeneity. Step 1, placement of FFPE CDX on capture areas of a Visium gene expression (GEX) slide. Step 2, H&E staining followed by brightfield microscopic imaging with ZEISS Axioscan 7 high-performance slide scanner (White Plains, NY). Step 3, permeabilize tissue and construct barcoded libraries with a final sample index PCR according to the manufacturer’s instructions. Step 4, short-read sequencing (Illumina NovaSeq) of barcoded libraries by Genewiz. Step 5, data analysis of tissue images and sequencing files in FASTQ format with Space Ranger run on Ubuntu 22.04 LTS–Thelio Mira-b3 by System76, Inc. (Denver, CO). The space ranger aggr pipeline aggregated data from replicate samples and from replicate regions. Loupe browser visualization software was accessed in a desktop application via Windows (Dell Optiplex 990).

#### FFPE sections

CDX sections (5 μm) were processed with the RNeasy FFPE kit for DV200 analysis. Replicate sections from *HLTF*^*-/-*^ and *HLTF*^*+/+*^CDX from ID *Hltf* KO mice were placed within fiducial frames of capture areas A,B and C,D respectively, on Visium GEX slide V11D13-089-A1.

#### GEX slide

each of 4 capture areas (6.5 x 6.5 mm each) inside fiducial frames (8 x 8 mm each) contain 5,000 gene expression spots (55 μm in diameter) with a distance of 100 μm between the centers of each spot (1–10 cells/spot). Visium for FFPE uses RNA-templated ligation (RTL) probes targeting the whole transcriptome. The assay captures probes via a capture sequence, e.g. poly-A for FFPE probes. Each gene expression spot has primers with a unique spatial barcode. Probes are designed against the entire human genome, each with primers that include Illumina TruSeq Read 1 (partial read 1 sequencing primer), 16 nt spatial barcode, 12 nt unique molecular identifier (UMI), and 30 nt poly(dT) sequence (captures ligation product). Spatially barcoded, ligated products were released from the slide, and harvested for qPCR with KAPA SYBR Fast qPCR master mix. The threshold for determining the Cq value for each sample was set along the exponential phase of the amplification plot at ~25% of the peak fluorescence value with QuantStudio 12 K Flex real-time PCR system (ThermoFisher Scientific). Sample index sets were selected to distinguish each of the 4 samples in a multiplexed sequencing run. Samples were amplified using Ilumina-compatible indexing primers, cleaned up with SPRIselect reagent, and bi-directionally sequenced.

#### Human probe set

Visium Human Transcriptome Probe Set v1.0 contains 19,144 gene ids targeted by 19,902 probes. Gene ids (1,201; 6.3%) targeted by 1,272 probes were excluded by default due to predicted off-target activity to a different gene. As a result, 17,943 gene_ids (targeted by 18,630 probes) were present in the final filtered output. During data analysis, read 2 sequences were mapped against the reference human genome (GRCh38/hg38) and read 1 sequences were used for UMI filtering to obtain spatial information.

#### Sequencing

Illumina NovaSeq NGS was performed at GeneWiz. Unique dual indexing—unique identifiers on both ends of the sample—allowed for an increase in the number of samples sequenced per run. Sequencing depth, a minimum of 50k read pairs per spot covered with tissue, was calculated by estimating the percent of capture area covered by the tissue section based upon the H&E brightfield image. Actual values are provided in Table 3.

**Table 3:** Statistics for spatial transcriptomics outcome for Visium_FFPE_*HLTF*^-/-^CDX in ID *Hltf* KO mice (A and B) and *HLTF*^*+/+*^CDX in ID *Hltf* KO (C and D).

ID	DV200	# of spots under tissue	Mean reads/spot	Median genes/spot	# of reads	Validated barcodes	Sequencing saturation	Genes Detected
A	94.42	3,159	277,048	5,107	875,195,352	98.1%	89.2%	17,089
B	94.42	2,757	208,484	2,629	574,790,269	98.0%	93.3%	15,883
C	92.64	2,018	309,788	9,724	625,152,273	98.5%	61.9%	17,299
D	92.64	2,206	341,062	9,362	752,382,625	98.4%	73.5%	16,386

100% of UMIs valid for each sample

### Bioinformatics analysis

Visium Spatial Gene Expression Software Suite includes Space Ranger and Loupe Browser. Spaceranger mkfastq demultiplexed the Illumina sequencer’s base call files (BCLs) for each flow cell directory into FASTQ files. Spaceranger count combined a brightfield microscope slide image and FASTQ files from spaceranger mkfastq and performed alignment, tissue detection, fiducial detection, barcode/UMI counting, and prepared a full resolution slide image for visualization in Loupe Browser. The pipeline used the Visium spatial barcodes to generate feature-spot matrices, determine clusters, and perform gene expression analyses. The pipeline used a probe aligner algorithm for FFPE tissues. Spaceranger aggr used the output of multiple runs of spaceranger count from related samples and aggregated their input, normalizing those runs to the same sequencing depth, and then recomputed the feature-barcode matrices and the analysis on the combined data. The aggr pipeline combined data from multiple samples into an experiment-wide feature-barcode matrix and analysis. Loupe Browser was used to interrogate significant genes, characterize and refine gene clusters, and perform differential expression analyses. Spatial transcriptomics data for *HLTF*^*-/-*^ and *HLTF*^*+/+*^CDX tumors in ID *Hltf* KO mice are accessible through NCBI’s Gene Expression Omnibus (GEO) SuperSeries accession number GSE234728, SubSeries GSE234517 (Spatial).

### Protein S-Glutathionylation (Pr-SSG)

Parallel analysis of Pr-SSG in *HLTF*^*-/-*^HCT116-Red-Fluc cells used for OCMI and resultant *HLTF*^*-/-*^CDX in ID *Hltf* KO mice was performed by Applied Biomics, Inc (Hayward, CA). Briefly: cell pellet (control) and CDX (n = 2 mice) were homogenized/sonicated (Polytron) in 4 volumes HENS buffer. Protein concentrations were determined (OD_280_) with NanoDrop One (ThermoFisher), adjusted to a final concentration of 2 μg/μl with HENS buffer and frozen for analysis.

Differential Pr-SSG between cell and tumor proteins was evaluated by large-format two-dimensional gel electrophoresis (Western blot) and two-dimensional difference gel electrophoresis (2D DIGE) in which cell (gel 1) and tumor proteins (gel 2) labeled with Cy2 (internal standard) were interrogated with mouse monoclonal anti-glutathione (0.5 μg/ml) and Cy5-labeled donkey anti-mouse IgG (1:2000 dilution). These two gels provided information on the difference in glutathionylation between cells and tumor (glutathionylation ratio). Difference in protein levels between the two samples (ratio) was determined by comparing Cy2 labeled cell proteins with Cy3 labeled tumor proteins by 2D DIGE (gel 3). The final glutathionylation ratio for each spot was obtained by adjusting the glutathionylation ratio by the protein ratio. 2D-DIGE guided the excision of 61 spots. Protein identification was based on peptide fingerprint mass mapping (using MS data) and peptide fragmentation mapping (using MS/MS data). MASCOT search engine was used to identify human and mouse proteins from primary sequence databases (SWISSProt). Posttranslational modification (glutathionylation) authentication was based on matrix-assisted laser-desorption ionization time-of-flight (MALDI-TOF/TOF).

## Results and discussion

**Fig 1:**
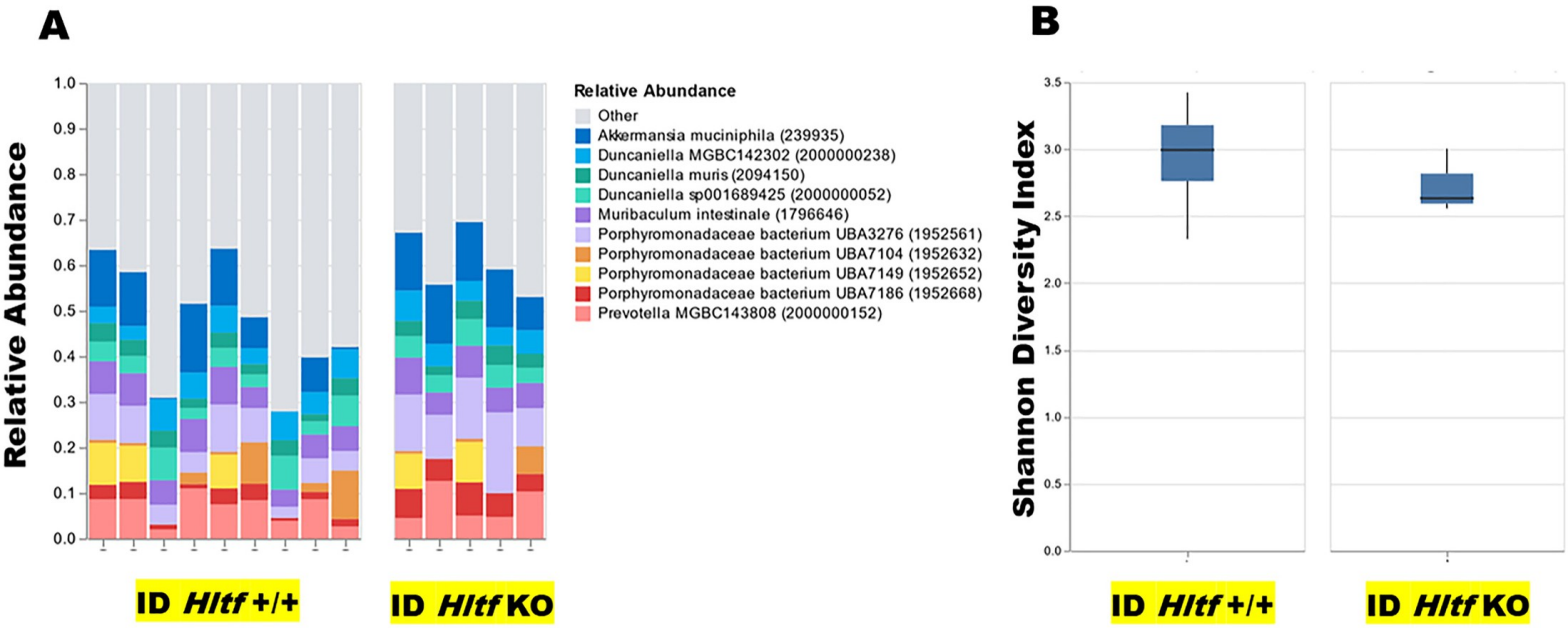
Cohort plots of taxonomic data. A. The relative abundance of each microbial species was estimated based on the depth and coverage of sequencing across every available reference genome. The top 10 species are provided in bar graph format for individuals subdivided into two cohorts, i.e. ID male mice that were either *Hltf* +/+ (control) or *Hltf* KO. B. The Shannon Diversity Index was used to measure the diversity of taxa within the samples and between the cohorts. Box-and-whisker plots summarize numerical taxonomic (family) data based on quartiles. The inner quartile range < 2 indicates the values are not significantly different despite the differences in median values of 2.99 and 2.63, respectively, for *Hltf* +/+ and *Hltf* KO.

The goal of the first experiment was to characterize the gut microbiome component of the TME [17] because it can influence CRC susceptibility and advancement. Cohoused mice share their microbial communities by coprophagy. By sequencing high molecular weight genomic DNA—which captured the true microbial diversity of stool samples from age-matched *Hltf*^+/+^ (n = 9) and global *Hltf* KO (n = 5) ID *Rag2-/IL2-* males—we were able to eliminate the gut microbiome as a variable in this study (Fig 1). No significant differences were observed in the microbial species in the two groups of male mice. More importantly, the microbial species were all anaerobic and could be eliminated because GSH-associated metabolism occurs primarily in aerobic bacteria [18].

### *HLTF*-deleted isogenic HCT116-Red-FLuc cells

**Fig 2:**
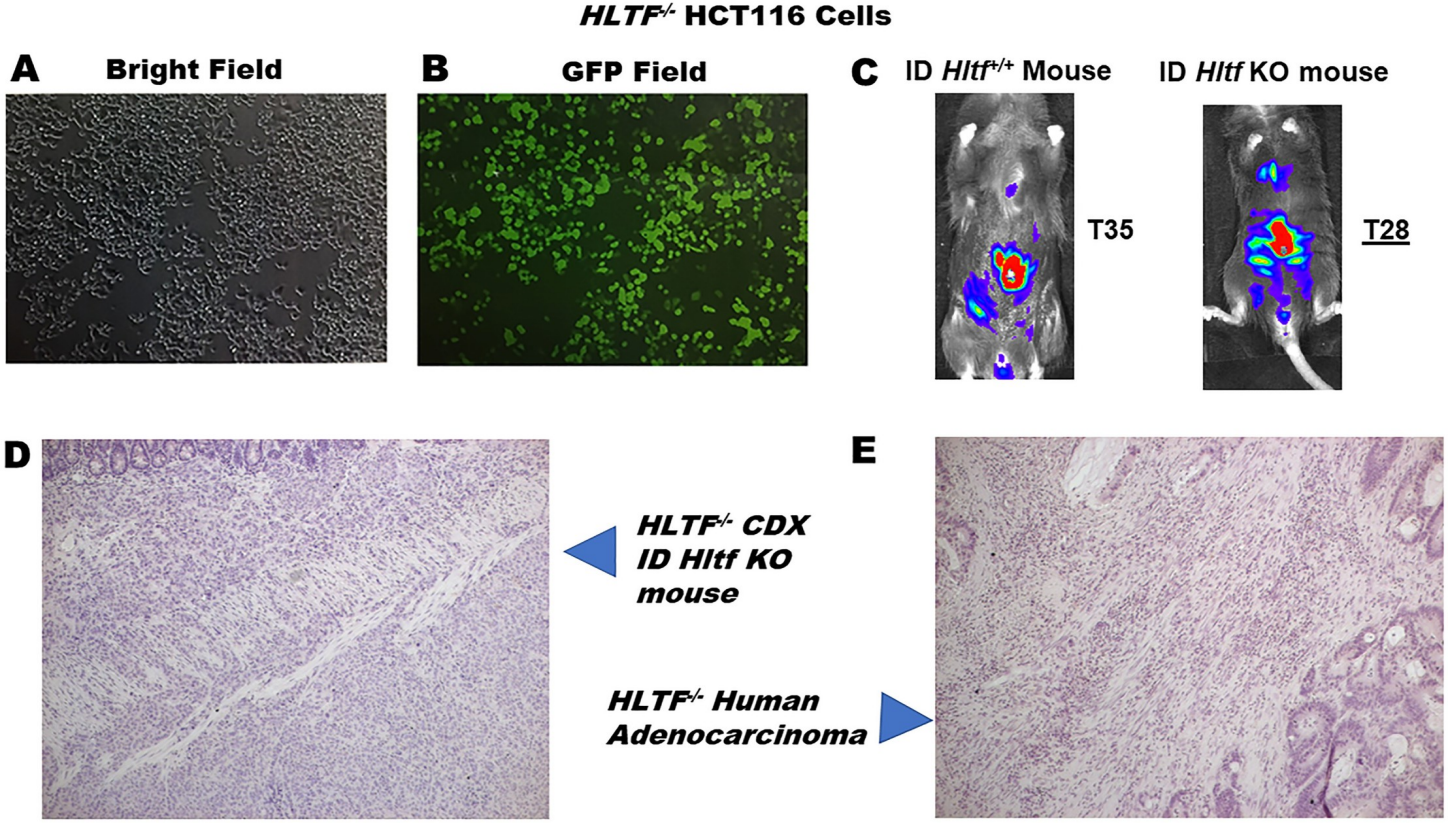
*HLTF*^-/-^HCT116-Red-FLuc cells, *HLTF*^*-/-*^CDX/TME model and human primary adenocarcinoma of male cecum (T4aN2bM1). Adherent, puromycin-resistant *HLTF*^-/-^HCT116-Red-FLuc cells were monitored with bright field (A) microscopy. The polyclonal nature of the cell line was evident when monitored for green fluorescent protein (GFP) expression (B). The shorter survival time, i.e. termination (T) at 28 vs 35 days for ID *Hltf* KO mice with *HLTF*^*-/-*^CDX vs *HLTF*^*+/+*^CDX, coincided with invasion and metastasis as monitored with BLI (C). Photomicrographs highlight negligible HLTF immunostaining throughout the tumor and TME in the mouse model (D) comparable to the human adenocarcinoma of the cecum (E). Photomicrographs at 10X magnification.

The goal of the second experiment was to characterize the HCT116-Red-FLuc isogenic cell line. The shRNA-silenced stable isogenic cell line was expanded and monitored for GFP expression (Fig 2). When grown in McCoy’s 5a Modified Medium supplemented with 10% fetal bovine serum and puromycin (2 μg/mL) *HLTF*^-/-^HCT116-Red-FLuc cells averaged the same doubling time (16 hours), shared the same morphology in culture, and had the same kinetic curve for their bioluminescence signal post-Luciferin injection (peak time 10–20 minutes) as parental *HLTF*^+/+^HCT116-Red-FLuc cells. Cells were further characterized as controls in the 2D-DIGE MALDI-TOF/TOF mass spectrometry experiments.

### Experimental timeline ensured mouse survival

The goal of the third experiment was to establish primary xenografts with *HLTF*^-/-^HCT116-Red-FLuc cells in ID *Hltf* KO mice. When Cespedes et al [19] developed the OCMI model, HCT116 cells in Swiss Nu/Nu mice produced highly aggressive, disseminating stage IV tumors that caused death in 39+4 days. We implemented the model to confirm the negative correlation between *HLTF* expression in tumor cells and survival in mice previously identified with a non-metastatic AOM/DSS model [20]. ID *Hltf* KO mice are associated with a worse prognosis and are 5-times more likely to die during the treatment protocol compared to ID *Hltf*^*+/+*^ mice [10]. Establishing a survival timeline at 28 days for ID *Hltf* KO mice inoculated with *HLTF*^-/-^HCT116-Red-FLuc cells (Fig 2) ensured all mice survived to the end of this study.

### *HLTF* expression is negatively associated with survival in human CRC

The negative correlation between *HLTF* expression and the progression of CRC is well documented in humans [5, 21–25]. With early-stage CRC, *HLTF* is detectable in tumor cells but negligible in the TME. With progression, epigenetic silencing of *HLTF* in tumor cells coincides with negligible *HLTF* expression in the TME. The *HLTF*^-/-^CDX/TME model in this study recapitulates end stage disease progression. As shown in Fig 2, the absence of HLTF protein expression is indistinguishable from the moderately differentiated adenocarcinoma of cecum from a 54-year-old male (T4aN2bM1). The patient’s primary tumor invaded through the muscularis propria and penetrated to the surface of the visceral peritoneum (T4a). Metastasis was found in a total of 19/27 regional lymph nodes (N2b), with spread to liver, lung, and omentum (M1). All CDX attached to the parietal peritoneum (T4b) as with end stage human CRC (peritoneal carcinomatosis).

### Comparative CDX transcriptomics

**Fig 3:**
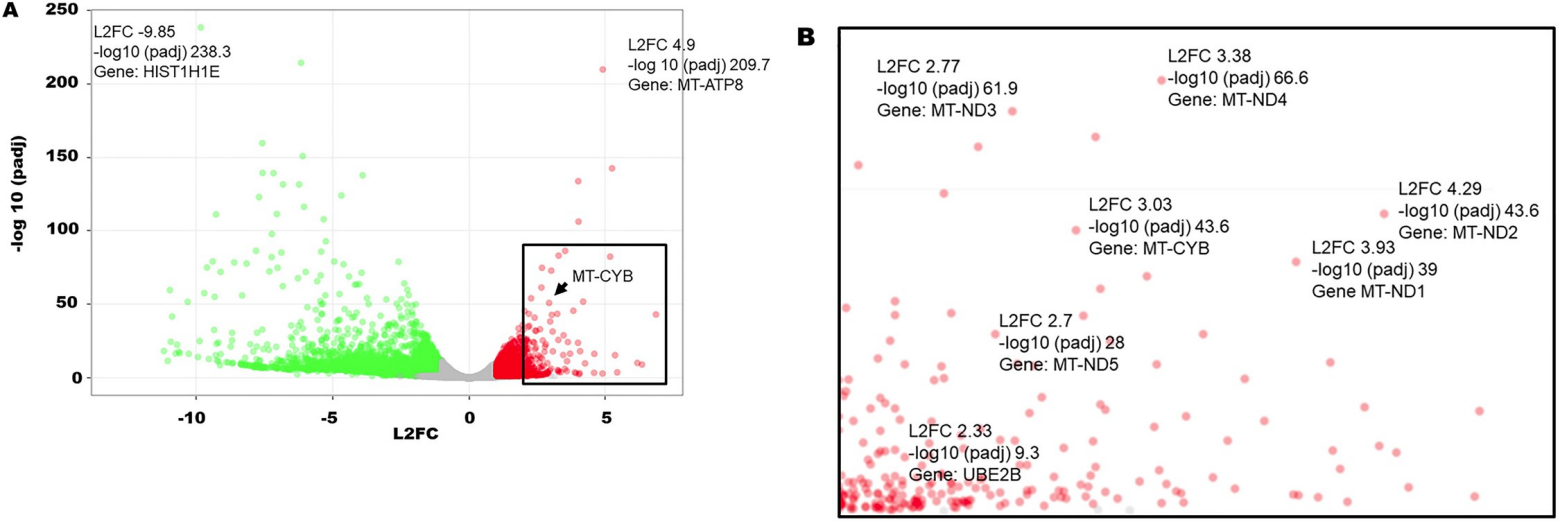
The global transcriptional change across the groups compared was visualized by a volcano plot (A) with a boxed region expanded in the inset (B). Each data point in the scatter plot represents a gene. The L2FC of each gene is represented on the x-axis and the log10 of its padj is on the y-axis. Up-regulated genes padj value < 0.05 and a L2FC > 1 are indicated by red dots. Down-regulated genes padj value < 0.05 and a L2FC < -1 are indicated by green dots. Examples of up- (*MT-ATP8*) and down- (*HIST1H1E*) regulated genes are labeled for reference along with up-regulated expression of the *MT-CYB* gene in the volcano plot (A) and in the inset (B) with an exploded view of additional genes.

In the fourth experiment, DESeq2 was used for comparison gene expression analysis between *HLTF*^*-/-*^ and *HLTF*^*+/+*^CDX from ID *Hltf* KO mice. Genes with padj < 0.05 and absolute L2FC > 1 were called as differentially expressed (DE) genes. There was a total of 1,599 upregulated genes and 3,493 downregulated genes (Fig 3). Following species-specific transcriptome alignment, DE genes were clustered by their gene ontology (GO). Enrichment of GO terms for *HLTF*^*-/-*^CDX vs *HLTF*^*+/+*^CDX in ID *Hltf* KO mice was tested using Fisher exact test (GeneSCFv1.1-p2).

**Fig 4:**
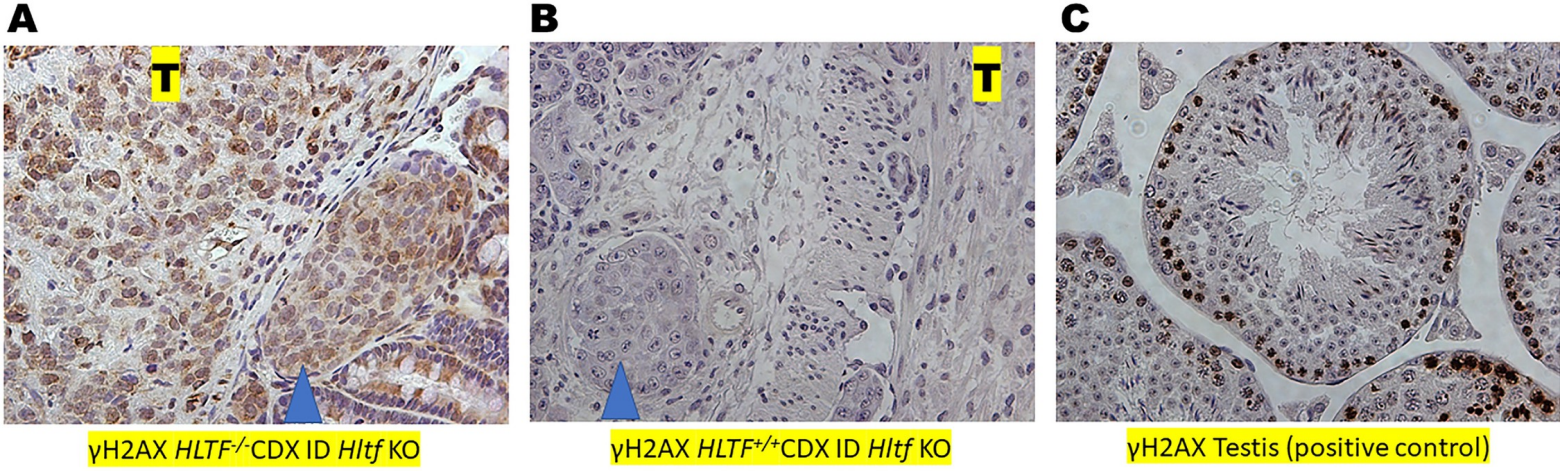
Immunolabeling with anti-γH2AX. Extensive γH2AX labeling in *HLTF*^*-/-*^CDX ID *Hltf* KO mice from endogenous DNA damage (A) strongly contrasts with the absence of γH2AX labeling in *HLTF*^*+/+*^CDX ID *Hltf* KO mice (B). CDX tumors (T) as well as tumor cells in lymphatic intravascular niches (blue arrow heads) of the mouse TME are labeled. (C) γH2AX labeling in mouse testis during meiosis (positive control) occurs in all intermediate and type B spermatogonia (last mitotic cells) and in preleptotene through zygotene spermatocytes during normal spermatogenesis. Panels A-C, 40X magnification.

GO analysis identified transcriptional activation (padj 0.04649) of 39 genes involved in the cellular response to a DNA damage stimulus (GO:0006974; Fig 3) coincident with γH2AX detection in *HLTF*^*-/-*^CDX ID *Hltf* KO mice (Fig 4). Human HLTF is a multidomain protein that safeguards genomic integrity by exerting independent functions in nucleotide excision repair (NER) and post replication repair (PRR) including translesion synthesis [25, 26]. While there is a paucity of information regarding *HLTF* loss of function in NER and PRR during *in vivo* tumorigenesis, DNA damage from double stranded breaks was quickly followed by phosphorylation of Ser-139 of the histone variant H2AX forming γH2AX [27]. Detection of nuclear foci of newly phosphorylated protein—a reliable marker of DNA damage initiation and resolution [28]—was accompanied by transcriptional activation (padj 0.00588) of 43 genes involved in DNA repair (GO:0006281; Fig 3).

### Oxidative stress and HIF-1α

**Fig 5:**
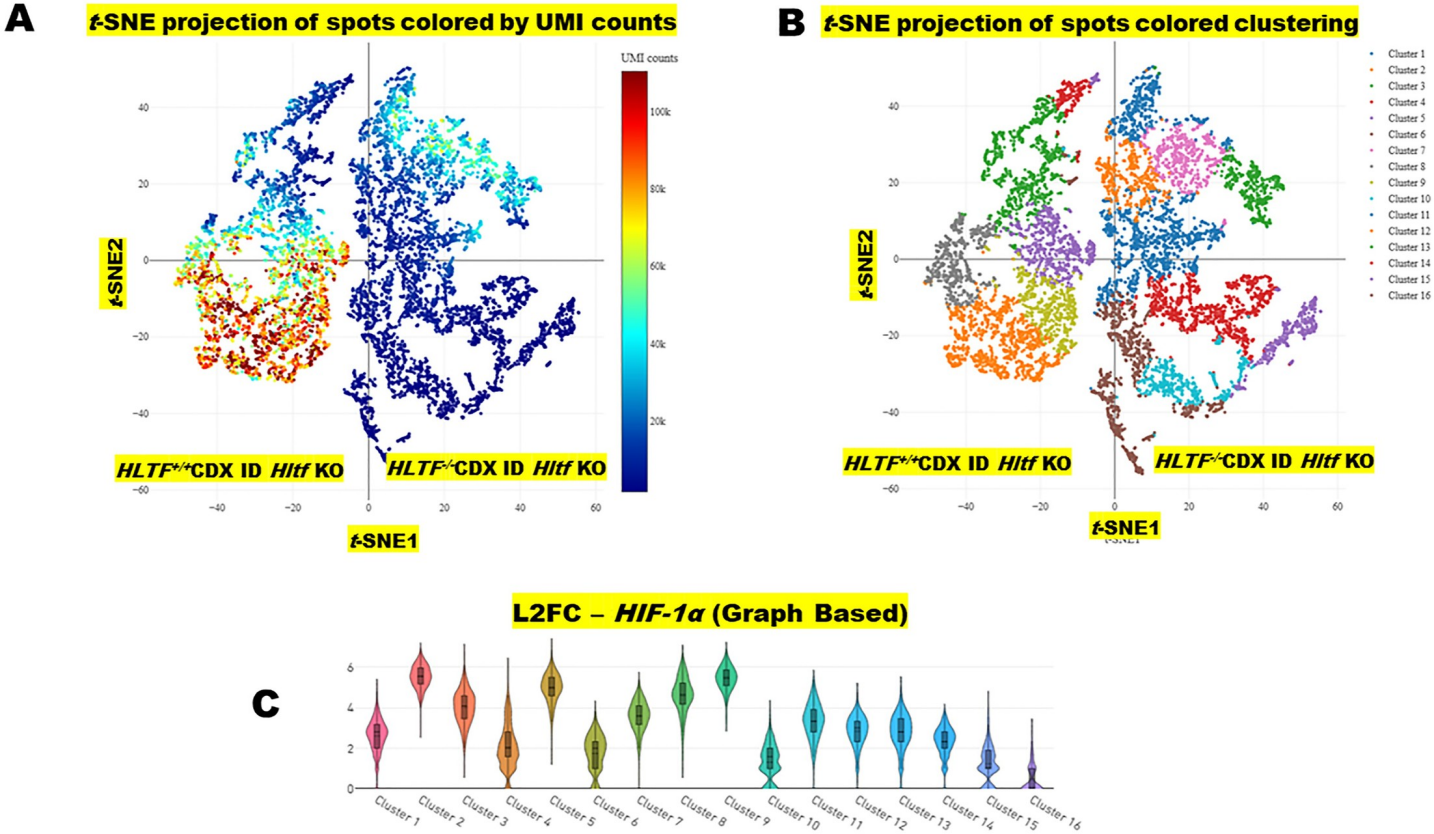
*t*-SNE projection of spots and a violin plot. Principle component analysis (PCA) was run on the normalized filtered feature-barcoded matrix to reduce the number of feature dimensions prior to clustering. For gene expression after PCA, *t*-distributed neighbor embedding (*t*-SNE) was run to visualize spots in a 2-D space, followed by clustering to group spots with similar expression profiles. *t*-SNE colored by UMI counts per spot (A) and spots by clustering (B) are shown for the entire data set. Comparative expression (C) analysis revealed variability in the spatiotemporal distribution of *HIF-1a* expression across 16 clusters for the entire data set.

Excessive production of reactive oxygen/nitrogen species (ROS/RNS) causes oxidative stress that has been implicated in the development and progression of CRC [29]. Mitochondrial dysfunction results from oxidative stress [30]. Transcription factor hypoxia inducible factor-1α (HIF-1α) mediates the mitochondrial response to oxidative stress [31] via two mechanisms [32, 33]. One is transcriptional activation in response to hypoxia, and the second is by a GSH adduct on human Cys520 (mouse Cys533) that stabilizes HIF-1α protein. Hltf is a transcriptional regulator of murine hypoxia inducible factor-1α (*Hif-1α*) in heart [14]. We previously reported [10] *HIF-1α* gene transcription in *HLTF*^*+/+*^CDX ID *Hltf* KO mice was unaffected by the presence or absence of *Hltf* in the TME. In the fifth experiment, we show deletion of *HLTF* from the tumor and the TME had no effect on total *HIF-1α* transcription or on its downstream targets. Neither the hypoxia-responsive genes involved in angiogenesis (*VEGF-A*, *PIGA*, *ANG2*, *PDGF-B*) nor the glycolytic enzymes (*ALDA*, *LDHA*, *PGK1*, *PKM*) with hypoxia response elements were transcriptionally activated. Cluster experiments from spatial transcriptomics allowed us to visualize *HIF-1a* throughout the tumors (Fig 5). The data set contained 88.7% of the reads in spots under tissue, encompassing 17,998 unique genes, and approximately 5,909 unique genes were identified per spot. Additional information about the spatially resolved dataset is provided in Table 4. The *t*-distributed neighbor embedding (*t*-SNE) projection plots—a statistical method for understanding high-dimensional datasets—identified UMI counts and 16 distinct clusters that strongly recapitulated CDX histology (Fig 5).

**Table 4:** Summary of site-specific S-glutathionylation identified by 2D-DIGE MALDI-TOF/TOF mass spectrometry. Pr-SSG in *HLTF*^*-/-*^CDX/TME.

Spot ID	Maldi well	Protein	Gene	Final adj WB ratio	Pr-SSG site	% identity Hu/Mu
14	F5	ENOA1	ENO1	2.5	Cys^119^	94.47
24	E7	PGAM1	PGAM1	9.5	Cys^55^	99.61
26	E8	PGAM1	PGAM1	17.1	Cys^55^	99.61
37	I14	HSP60	HSPD1	2.7	Cys^447^	97.56
43	F17	PGK1	PGK1	0.4	Cys^379/380^	97.84
44	I18	ANXA1	ANXA1	1.8	Cys^263^	87.57

### OXPHOS and Pr-SSG

**Fig 6:**
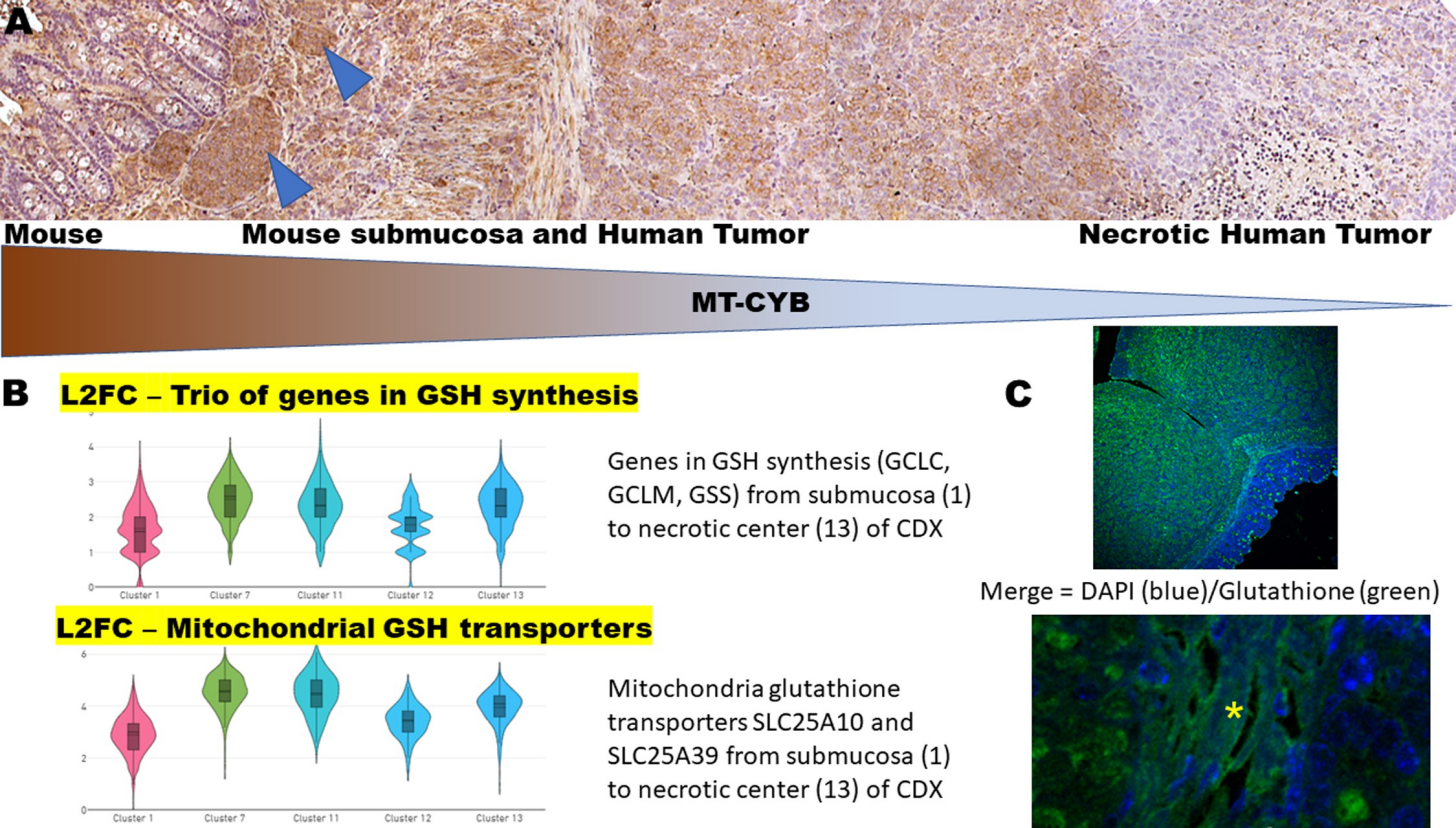
Indicators of OXPHOS and glutathione biosynthesis in *Hltf*^*-/-*^CDX model. (A) Photomicrograph emphasizes gradient of positive immunostaining for MT-CYB in tumor cells of the *Hltf*^*-/-*^CDX including tumor cells in lymphatic intravascular niches (blue arrow heads) of the mouse TME. 10X magnification. (B) Violin plots from spatial transcriptomics depicts regional gene expression. Each violin plot is a composite of genes. The top plot is for the trio of genes in GSH synthesis, i.e., the rate-limiting enzyme glutamate cysteine ligase (GCL), which is composed of catalytic (GCLC) and modifier (GCLM) subunits, and GSH synthetase (GSS). The bottom plot shows gene expression for mitochondrial glutathione transporters, *SLC25A10* and *SLC25A39*. (C) S-glutathionylation was visualized with a biotin switch assay and confocal imaging, which allowed for optimal detection of S-glutathionylated proteins over potential background signals. For data presentation, the maximal projection confocal images obtained from a z stack (500 nm slice) using 10x (upper) and 60x (lower) objectives (oil) are shown. Cells were visualized using Nikon T1-E microscope with A1 confocal and images were taken using NIS software. Merged images were obtained with transmitted light and excitation at 405 nm (DAPI, blue-violet) nuclei and 488 (streptavidin-labeled, green) S-glutathionylated proteins. Human tumor and mouse endothelial cells (yellow asterisk) harbor S-glutathionylated proteins. The mouse endothelium is in the lamina propria that subtends the mucosal epithelium.

In the absence of *HIF-1α* activation, a total of 23 human genes in mitochondrial electron transport, NADH to ubiquinone (GO:0006120), were transcriptionally activated (padj 3.73E-05) in conjunction with 25 human genes (padj 9.83E-05) in the mitochondrial respiratory chain complex 1 assembly (GO:0032981). Human *MT-CYB* (also *CYTB*)—a mitochondrially encoded component of the ubiquinol-cytochrome c reductase complex (complex III or cytochrome b-C1 complex), a respiratory chain that generates an electrochemical potential coupled to ATP synthesis—was one of the genes transcriptionally activated (Fig 3) in *HLTF*^*-/-*^CDX/TME (L2FC 3.03; padj 1.7E-44). Immunolabeling of MT-CYB protein—a marker for OXPHOS—identified mitochondria in tumor cells in the *Hltf*^*-/-*^CDX and in the host lymphovasculature (Fig 6). These findings in our preclinical model are consistent with elevated *MT-CYB* expression as a prognostic marker for metastatic and advanced clinical stage disease [34].

Mitochondria are key generators of cellular reactive ROS [35] and they have multiple antioxidant strategies to neutralize ROS including glutathione (GSH; γ-glutamyl-cysteinyl-glycine), a low molecular weight antioxidant exclusively synthesized in the cytosol in two ATP-dependent steps [36]. Solute carrier family 7 member 11 (SLC7A11) also known as xCT mediates the uptake of extracellular cystine in exchange for glutamate [37]. Cystine is reduced to cysteine. In the first step, γ-glutamylcysteine is synthesized from L-glutamate and cysteine by glutamate cysteine synthetase (GCL). In the second step, glycine is added to the C-terminal of γ-glutamylcysteine by glutathione synthetase (GSS), which is also a potent antioxidant. In the CDX tumors, transcriptional availability of the *SCL7A11* gene is the same for *HLTF*^*+/+*^ and *HLTF*^*-/-*^CDX ID *Hltf* KO mice. SLC7A11-AS1, an overlapping *cis*-natural antisense transcript at the xCT gene locus that inhibits xCT expression was transcriptionally downregulated (L2FC -6.87; padj 3.86E-09) in *HLTF*^*-/-*^CDX.

Spatial transcriptomics provided an unprecedented view of region-specific human *GSH* gene expression and compensated for the loss of structure that resulted from tissue dissociation for RNAseq experiments. It enabled us to simultaneously document cancer cell location in the CDX histology sections, and view the combinatorial expression of genes in GSH synthesis within that morphological context (Fig 5). GSH diffuses across the outer mitochondrial membrane, is actively transported across the inner mitochondrial membrane, and concentrated in the matrix. In the human *HLTF*^*-/-*^CDX, *SLC25A10*, the mitochondrial glutathione transporter [38] was upregulated (L2FC = 1.22; padj 3.12E-06) commensurate with down regulation (L2FC = -1.2; padj 9.04E-07) of its transcriptional repressor, BTB and CNC Homology 1 (*BACH1*) [39]. Region-specific genes in GSH synthesis and the expression of genes for mitochondrial GSH transport overlap in *HLTF*^*-/-*^CDX (Fig 6).

In mouse TME a total of 22 genes in the glutathione metabolic process (GO:0006749) were upregulated (padj 0.025974). GSH plays a critical role in endothelial function especially vasodilation and paracellular permeability [40]. Glutathione also has a role in protein post-translational modification [PTM, 41]. S-glutathionylation is a reversible PTM on thiol groups in protein cysteine (Cys) residues. As shown in Fig 6, the biotin-switch assay with FFPE *HLTF*^*-/-*^CDX/TME showed protein S-glutathionylation in human tumor cells and endothelium of mouse lymphovasculature. These data support the conclusion that increased availability of glutathione buffers oxidative stress, and drives tumor/endothelial cell proliferation and migration in the *HLTF*^*-/-*^CDX/TME model.

### Pr-SSG diverts glycolysis

**Fig 7:**
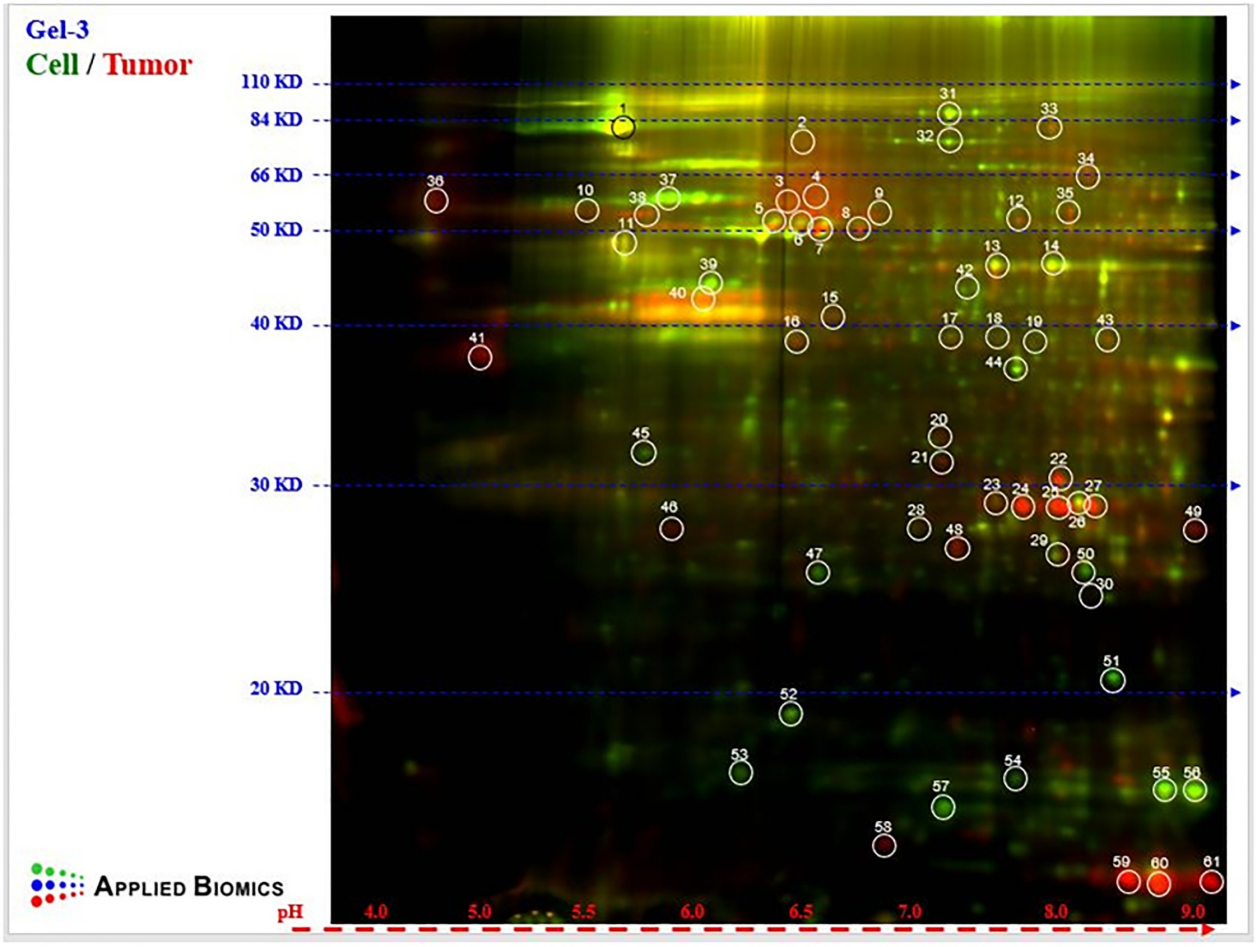
2D-DIGE gel electrophoresis. For purposes of data presentation, all 61 spots are labeled in the gel. The pH range 4 to 9 from isoelectric focusing is indicated at the X-axis (red horizontal line). Molecular mass markers (20–180 KD) are provided on the y-axis. All 61 differentially expressed proteins were subjected to MALDI-TOF/TOF analysis.

Hypoxia induced *S*-glutathionylation of HIF-1α on Cys^520^ (mouse Cys^533^) stabilizes the protein [33]. Thus, we directly tested the hypothesis that protein S-glutathionylation in *HLTF*^*-/-*^CDX/TME targeted Hif-1α protein in the sixth experiment. A total of 61 differentially expressed proteins were identified by 2D-DIGE comparison of glutathione-labeled proteins from *Hltf*^*-/-*^HCT116-Red-Fluc cells at the time of OCMI and resultant *HLTF*^*-/-*^CDX from ID *Hltf* KO mice (Fig 7 and S1 Raw_images). Final adjusted WB ratios for tumor proteins/cell proteins were calculated (S2). Protein authentication by MALDI-TOF/TOF led to the authentication of site-specific Pr-SSG of 6 proteins (Table 4). Fragments were omitted, and only protein and total ion C.I.% scores equal to 100% were included.

Neither human (Cys^520^) nor mouse (Cys^533^) HIF-1α is a target of S-glutathionylation in this CDX model. Further use of MALDI-TOF/TOF followed by species-specific data analysis identified highly conserved cysteines in identical peptide fragment ion spectra (S2) from human and mouse, leading us to conclude humans and mice share the same Pr-SSG protein targets in this *HLTF*^*-/-*^CDX/TME model. Protein conservation is shown in Table 4.

**Fig 8:**
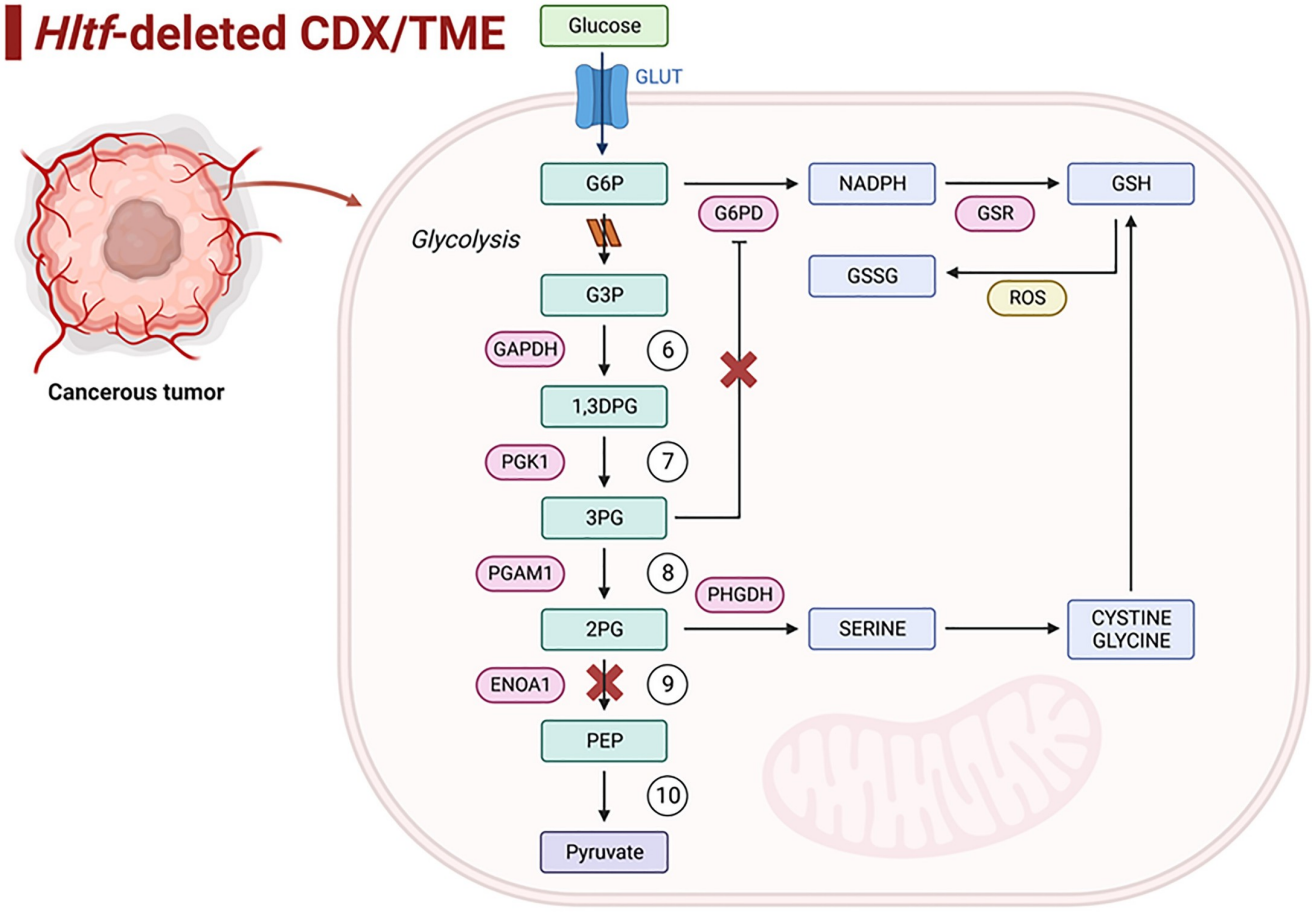
Diversion of energy releasing steps in glycolysis by Pr-SSG. The diagram shows energy releasing steps 6–10 of the glycolysis pathway. The inhibitory effects of Pr-SSG begin with the inhibition of glycolysis resulting from the loss of ENOA1 enzymatic activity. The resultant accumulation of 2PG activates PHGDH to provide feedback control of 3PG promoting antioxidant defense and cell proliferation. Created with Biorender.com.

Importantly, three of five Pr-SSG protein targets are glycolytic enzymes that control reversible steps 7, 8 and 9 in the 10-step glycolysis pathway (Fig 8). Glycolytic intermediates including glucose-6-phosphate (G6P) can be diverted into the oxidative pentose phosphate pathway to produce the antioxidant NADPH. Glyceraldehyde 3-phosphate dehydrogenase (GAPDH), the enzyme that catalyzes step 6, was not targeted for site-specific Pr-SSG thus avoiding programmed cell death. S-glutathionylation of GAPDH Cys^247^ shuttles GAPDH to the nucleus where it transfers the glutathione to Sirtuin-1 (SirT1) during SirT1-p53-mediated apoptosis [42].

Phosphoglycerate kinase (PGK1) and phosphoglyceromutase (PGAM1) are redox sensitive and exposure to glutathione stabilizes them (prevents inactivation) presumably via the newly identified S-glutathionylation sites reported here. PGK1 is a highly conserved so-called moonlighting protein because of its involvement in varied cellular processes as a protein kinase [43]. As the first ATP generating enzyme in glycolysis, PGK1 catalyzes the conversion of 1,3-diphosphoglycerate (1,3-DPG) to 3-phosphoglycerate (3PG), and PGAM1 catalyzes the interconversion of 3PG to 2PG.

**Fig 9:**
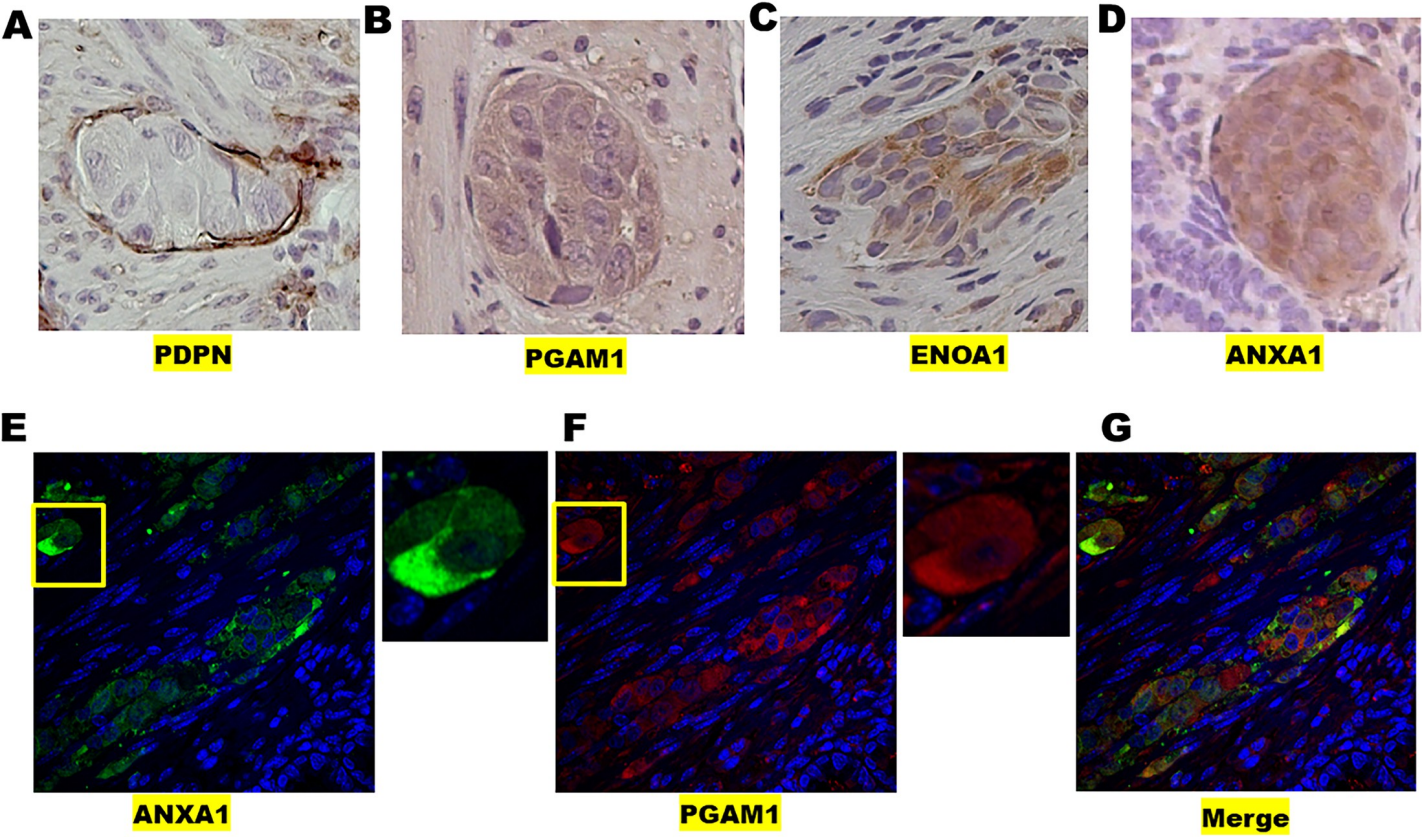
Protein localized in *HLTF*^*-/-*^CDX/TME. Light micrographs show positive immunostaining for PDPN (A), a lymphatic-specific endothelial cell marker, in companion with PGAM1 (B), ENOA1 (C) and ANXA1 (D) in tumor cells. 40X magnification. ANXA1 (E) and PGAM1 (F) colocalize (G) in tumor and endothelial cells in lymphatic intravascular niches. A polarized extravascular tumor cell with redistribution of ANXA1 and PGAM1 into a posterior uropod protrusion (yellow insets) is engaged in amoeboid migration, a hallmark of rapid migration [51]. The maximal projection of confocal images obtained from a z stack (500 nm slice) using a 60x objective (oil) are shown. Overlay images were obtained with transmitted light and excitation at 405 nm (DAPI, blue-violet), 555 nm (ANXA1, green) and 647 nm (PGAM1, red).

PGAM1 protein was the strongest candidate for further experimentation because its expression in *HLTF*-deleted CDX/TME had the highest (>5.0) final WB ratio indicating Pr-SSG occurred in cells of the CDX rather than the cultured cells from which it was derived. *PGAM1* was also transcriptionally activated (L2FC 1.81; padj 1.59E-08) in *HLTF*^*-/-*^CDX compared to *HLTF*^*+/+*^CDX from ID *Hltf* KO mice. Upregulation of PGAM1 is consistent with its upregulation in several human cancers [44]. PGAM1 immunolocalized to tumor and endothelial cells in the *HLTF*^*-/-*^CDX/TME (Fig 9).

3PG and 2PG are allosteric regulators of glycolytic branching pathways [45]. 3PG inhibits 6-phosphogluconate dehydrogenase (G6PD) in the pentose phosphate pathway. 2PG activates phosphoglycerate dehydrogenase (PHGDH) to provide feedback control of 3PG. PHGDH is the rate-limiting step in the *de novo* serine biosynthesis cascade that provides precursors for GSH synthesis and production of NADPH [46]. PHGDH was not targeted for site-specific Pr-SSG in our *HLTF*^*-/-*^CDX/TME model. This suggests PHGDH is functional because covalent modification of PHGDH Cys^295^ inactivates this enzyme [47].

The penultimate step in glycolysis—the conversion of 2PG to phosphoenolpyruvic acid (PEP)—is catalyzed by the rate-limiting enzyme, alpha enolase (ENOA1), another moonlighting enzyme [48]. Because ENOA1 is overexpressed in numerous different types of cancer, it is a target for therapeutic intervention. Glycolysis is significantly reduced (OXPHOS is rescued) when Pr-SSG of ENO1A Cys^119^ causes enzyme inactivation [49, 50]. Resultant 2PG accumulation activates PHGDH to deplete excess 3PG, thereby promoting serine synthesis and preventing 3PG-induced inhibition of NADPH production. ENOA1 immunolocalizes to tumor and endothelial cells in the *HLTF*^*-/-*^CDX/TME (Fig 9).

The Pr-SSG target, Annexin A1 (ANXA1, Cys^263^), was neither transcriptionally activated nor did it have a high final WB ratio (Table 4). Nevertheless, mouse ANXA1 is a known target for Pr-SSG at Cys^324^ with a putative role in redox signaling [52]. Pr-SSG at Cys^263^ in this *HLTF*^*-/-*^CDX/TME model suggests functional importance as does immunolocalization of ANXA1 protein in tumor and endothelial cells (Fig 9).

Independent of its metabolic activity, PGAM1 interacts with α-smooth muscle actin (ACTA2), and ANXA1 is linked to the actin cytoskeleton. Importantly, glutathionylated actin (GS-actin) on Cys^374^, a site conserved in all actin isoforms [53], was not a target in this *HLTF*^*-/-*^CDX/TME model. GS-actin has altered mechanical properties *in vitro* due to decreased polymerization G- to F-actin. Otherwise stated, deglutathionylation on Cys^374^ increases the rate of actin polymerization and cell motility [53]. Co-localization of PGAM1 and ANXA1 in invading lymphatic intravascular cancer cells and extravascular amoeboid cancer cells (Fig 9) was confirmed by laser scanning confocal microscopy. Front-rear asymmetry is characteristic of migrating lymphocytes [54]. However, the genetic background of the CDX model (*Rag2-/IL2*-double mutant) is completely alymphoid, which precludes confusion of cellular identify. Extravascular amoeboid cancer cells displayed aggressive metastatic directional movement consistent with an epithelial-to-mesenchymal transition [55].

PGAM1/ANXA1-positive tumor cells in intravascular niches corresponded to membership in spatial transcriptional Cluster 1 (Fig 5) concomitant with locoregional enrichment of the thymosin beta 4 X-linked (*TMSB4X*) gene (L2FC 1.86; padj 8.60e-32) whose secreted G-actin sequestering protein regulates actin dynamics. Transcriptional Cluster 1 was subepithelial and extended into the tumors resulting from circumferential cellular migration. Extravasculature PGAM1/ANXA1-positive tumor cells corresponded to membership in spatial transcriptional Cluster 14 (Fig 5) enriched for the epithelial membrane protein 1 *(EMP1*) gene (L2FC 2.99; padj 4.31e-27), a pro-metastatic factor responsible for metastatic recurrence in CRC. Transcriptional Cluster 14 subtends the epithelium independent of the tumor.

HSP60 is evolutionarily conserved. HSP60 typically resides in mitochondria where it maintains protein homeostasis via a protein folding apparatus with its co-chaperone HSP10 [56]. HSP60 monomers are composed of three structural domains designated apical, intermediate and equatorial, that are subjected to extensive modifications by TPMs [57]. Human and mouse HSP60 proteins are 97.56% identical, with three cysteine resides (Cys^237^, Cys^442^, and Cys^447^). Previously identified PTMs [57] related to nitration include S-nitrosylation at HSP60 Cys^237^ and S-guanylation at HSP60 Cys^440^. This is the first report of S-glutathionylation at HSP60 Cys^447^.

Results from this *HLTF*^*-/-*^CDX/TME model—show for the first time—that tumor cell recovery from DNA damage via robust transcriptional activation of DNA repair and OXPHOS genes coincided with a highly metastatic phenotype. Loss of *HLTF* function throughout the CDX/TME co-sponsored an amoeboid-associated TME at the tumor border. Cancer and endothelial cells of the TME share site-specific Pr-SSG signatures for 3 glycolytic enzymes that divert glycolysis in support of glutathione biosynthesis that prevents OXPHOS inhibition and promotes redox homeostasis.

Experimental advantages of this CDX model of CRC are validated by results from this study. First, by manipulating the genotype of the mice and HCT116 cells, the CDX model recapitulates the beginning and end of disease progression. Second, when human CRC is propagated in immune-deficient mice, not only does murine stroma and vasculature begin to replace the human equivalents immediately [58], but the human tumor induces reorganization of the quiescent, murine stroma into a pro-tumorigenic phenotype supporting tumor growth and metastasis [59]. We exploited this synergy in our preclinical CDX model. Third, the CDX model is predicated on direct orthotopic microinjection (OCMI) of HCT116 cells between the mucosa and the muscularis layers of the cecal wall known to replicate metastatic spread observed in advanced human CRC [19]. Fourth, RNAseq combined with species-specific mapping unambiguously differentiated gene expression in human tumor cells from cells of the mouse TME [10]. Fifth, a CDX histology section is entirely accommodated on the spatial transcriptomics array. As a result, human tumor cells were transcriptionally profiled as they interacted with the mouse TME, especially in lymphatic intravascular niches at the tumor margin. Sixth, protein posttranslational modifications were authenticated by using MS/MS data and MALDI-TOF/TOF through comparative analysis of tumor cells in culture with resultant CDXs.

One potential disadvantage of the CDX model is its development in an immune-deficient mouse. In defense of that approach, ~80–85% of CRC patients have so-called “cold tumors” meaning they do not have a strong immune response (do not express T-cells). Consequently, cold tumors are resistant to immunotherapy [60]. We posit our CDX model is relevant to immune-cold CRC. More importantly, spatial transcriptomics can be used to identify patients with active vs suppressed immune systems as a screening tool for therapy and to predict survival. Coupling mass spectrometry-based proteomics for FFPE [61] with spatial transcriptomics and immune profiling of FFPE could revolutionize our diagnostic/treatment approach to CRC.

## Conclusions

Collective results from the CDX model strongly support the conclusion that *HLTF*-deletion in cancer cells with resultant metabolic reprogramming—a hallmark of metastasis—is likely a prototypical example of cancer cells modifying their metabolic requirements in response to *Hltf*-deletion in the TME. The complete absence of HLTF from tumor cells and the TME produced the most aggressive metastatic phenotype with the shortest life expectancy. The next question is whether or not the complete absence of *HLTF* is a diagnostic marker for chemoresistance mechanisms in CRC because drug resistance is strongly correlated with glutathione synthesis [62].

## Supporting information

S1 Checklist NC3Rs ARRIVE guidelines checklist (fillable). (PDF)

S1 File Mass spec. (ZIP)

S1 Raw images (PDF)
