# Supplementary material for: Helicase-like transcription factor (HLTF)-deleted CDX/TME model of colorectal cancer increased transcription of oxidative phosphorylation genes and diverted glycolysis to boost S-glutathionylation in lymphatic intravascular metastatic niches
Source: PLoS One. 2023 Sep 8;18(9):e0291023. doi: 10.1371/journal.pone.0291023 (PMC10490896; doi:10.1371/journal.pone.0291023)
Supplement: S1 Raw images — (PDF) [file pone.0291023.s003.pdf]

Data Report: Project CHBE220713*

Project leader:	John Liao, PhD
Telephone number:	510-887-0889
Email address:	support@appliedbiomics.com
Company website:	www.appliedbiomics.com
Company address:	23785 Cabot Blvd. Suite 311-312<br>Hayward, CA 94545, USA

*The URL link to this report will remain active for 1 month

Experiment Summary

- **Gel layout**
  - Gel-1: Cell, WB with Anti-Glutathione Ab
  - Gel-2: Tumor, WB with Anti-Glutathione Ab
  - Gel-3: Cell / Tumor

- **Western Blot**
  - Primary antibody
    - Anti-Glutathione Ab: 0.5 μg/ml final concentration
  - Secondary antibody
    - Cy5-labeled Donkey anti-mouse IgG 1:2000 dilution

Western Images and Image Overlay

Gel_1

Cell | **Anti-Glutathione WB** | **Cell / Anti-Glutathione WB**

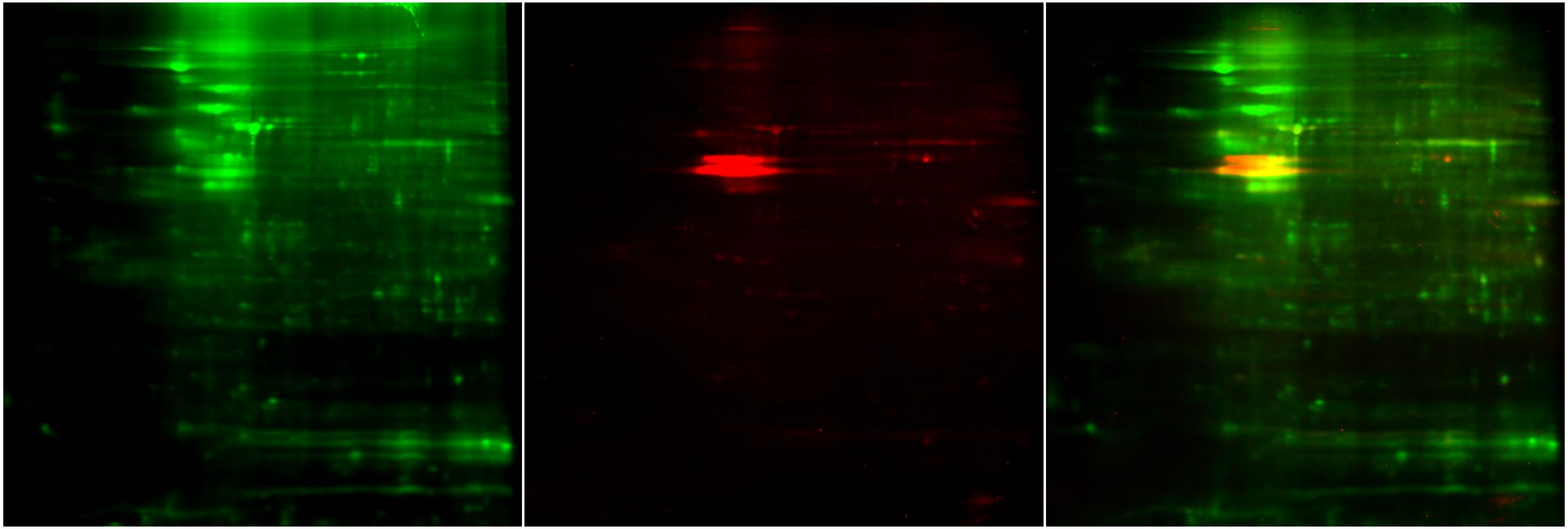

APPLIED BIOMICS

Gel_2

Tumor

Anti-Glutathione WB

Tumor / Anti-Glutathione WB

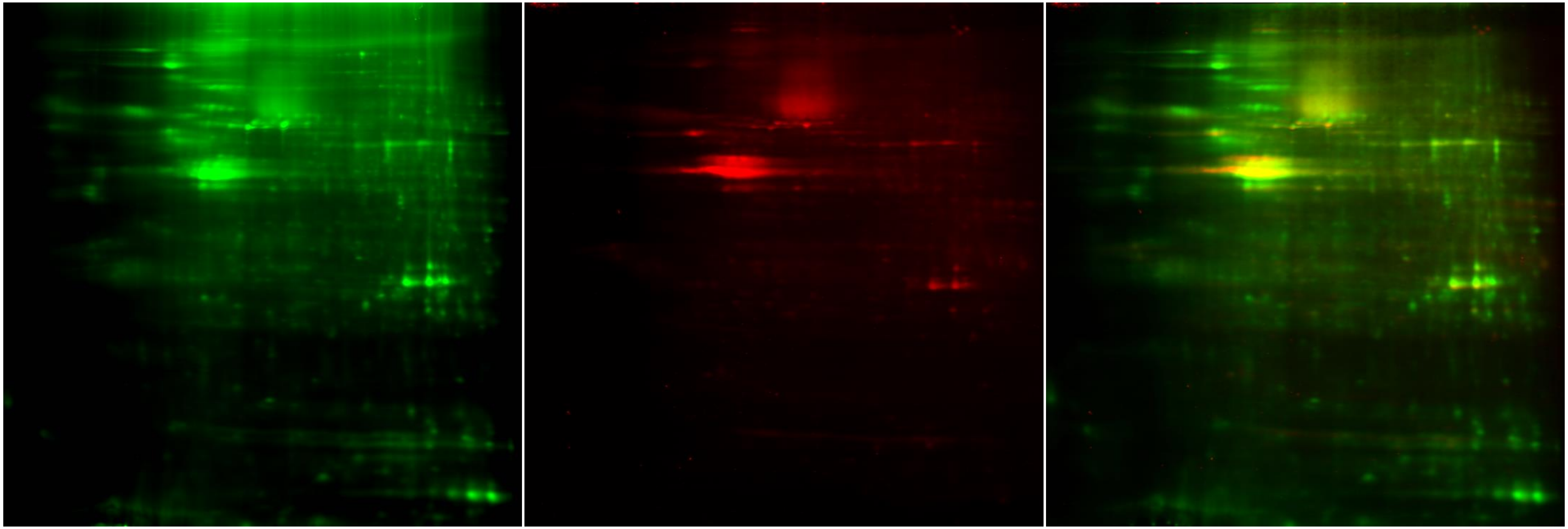

Applied Biomics

WB Images

Cell

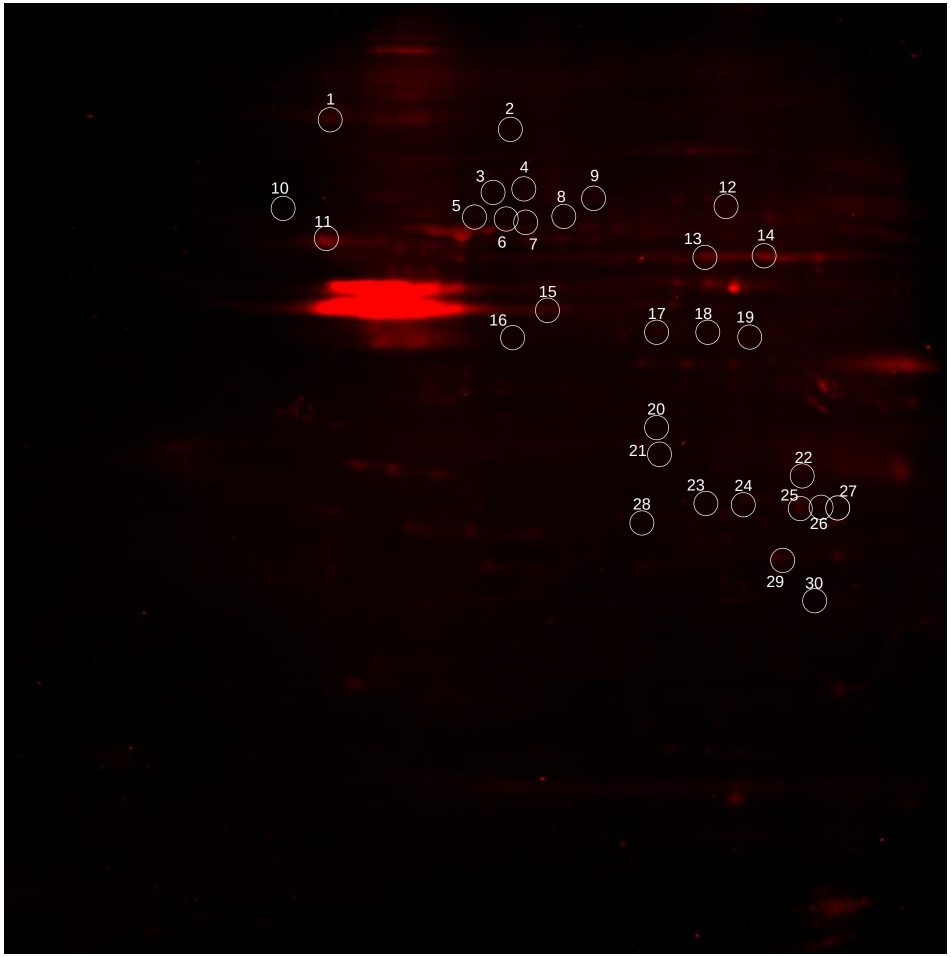

Tumor

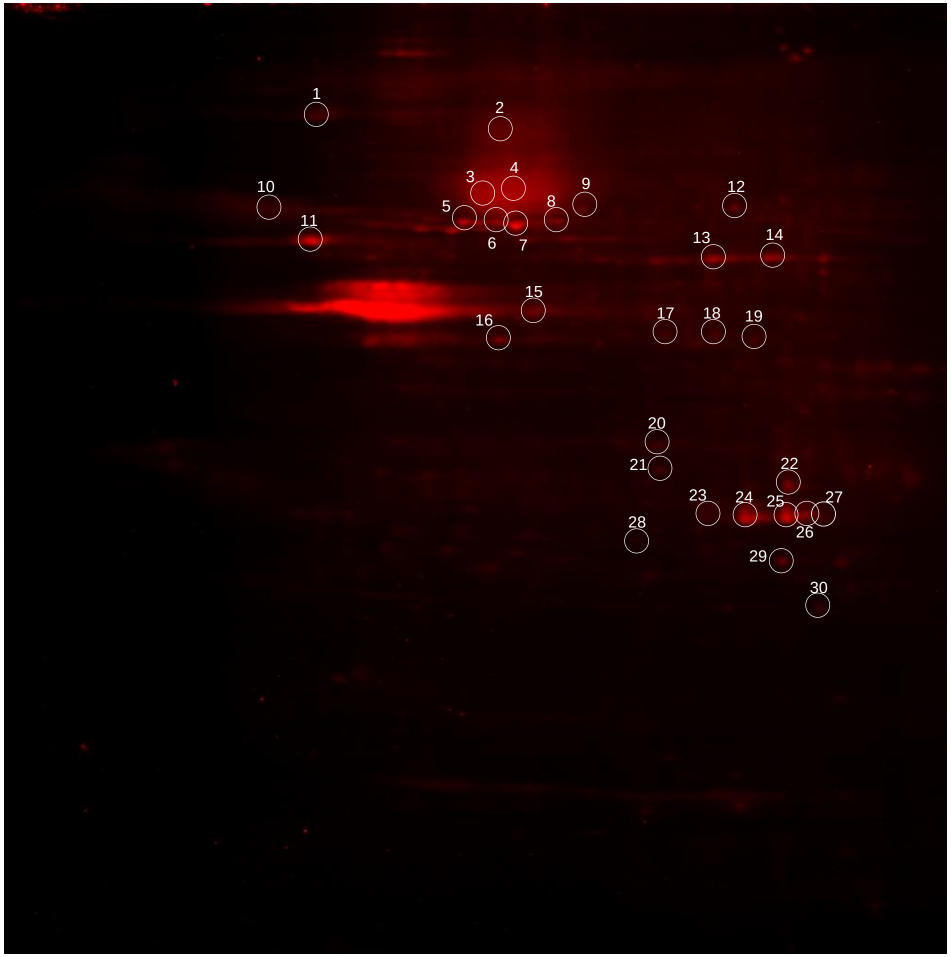

Applied Biomics

Protein / WB Image Overlay

Cell / Anti-Glutathione WB

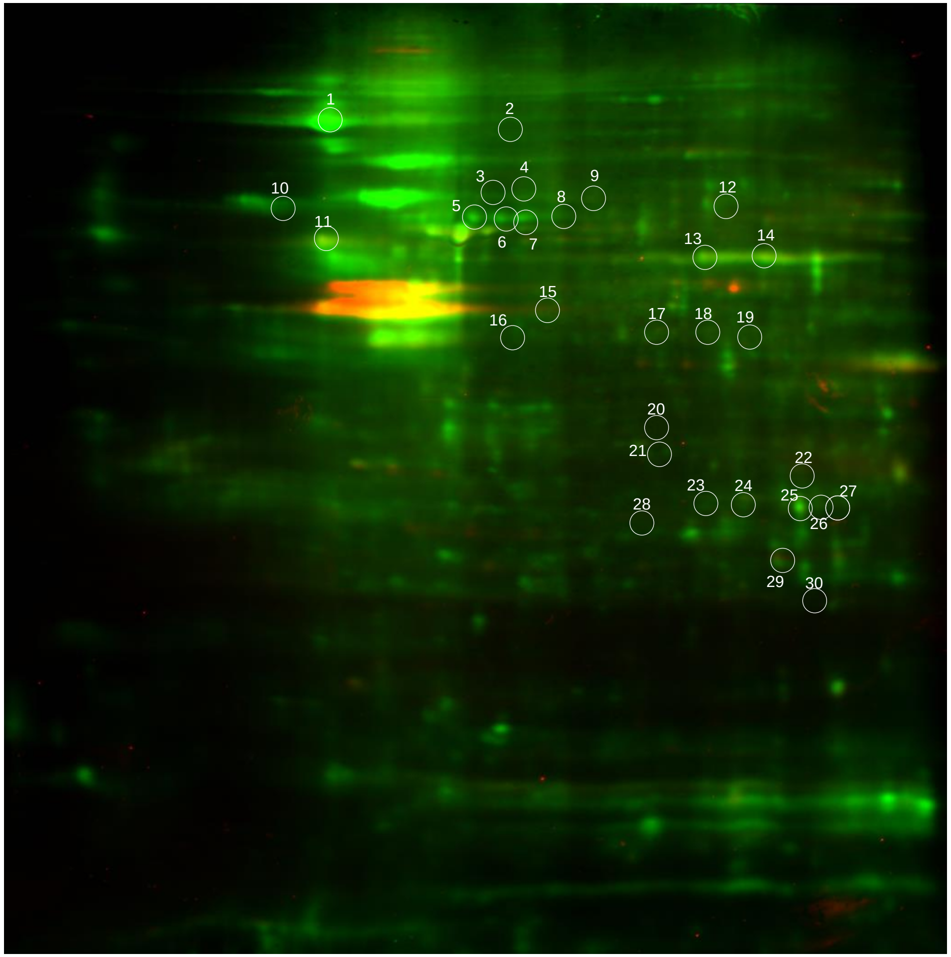

Tumor / Anti-Glutathione WB

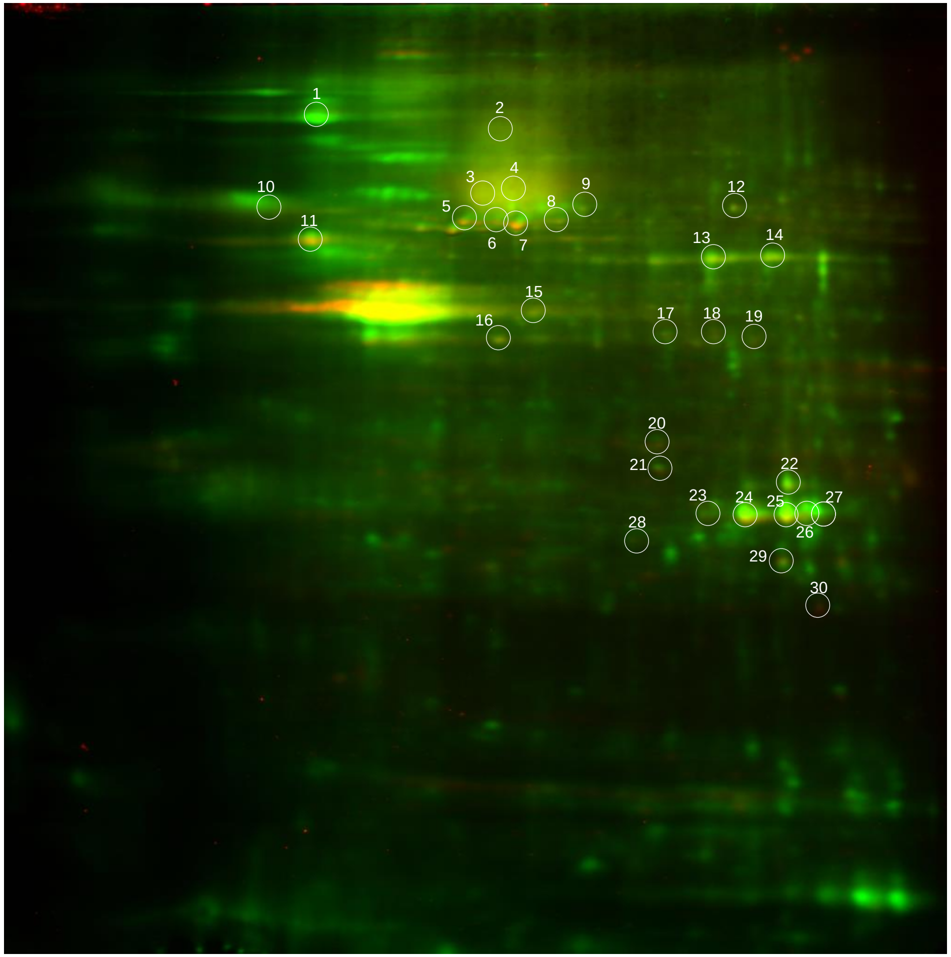

APPLIED BIOMICS

2D DIGE Images and Image Overlay

Gel-3

Cell | Tumor | Cell / Tumor

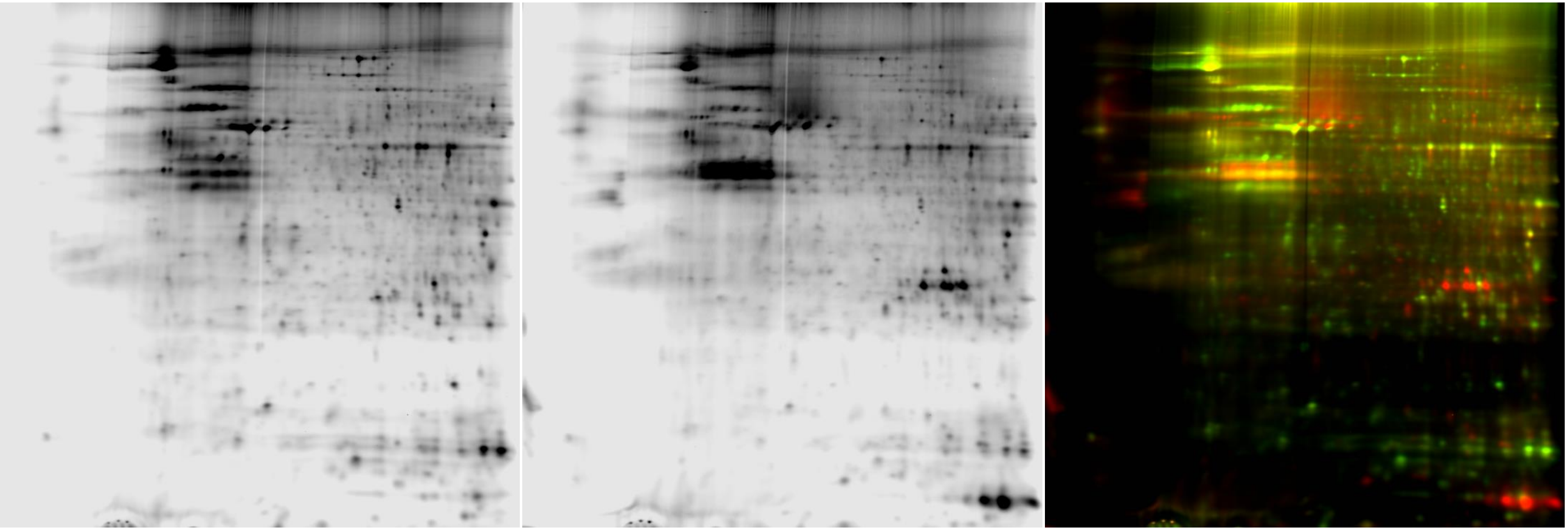

Applied Biomics

Gel-3

Cell

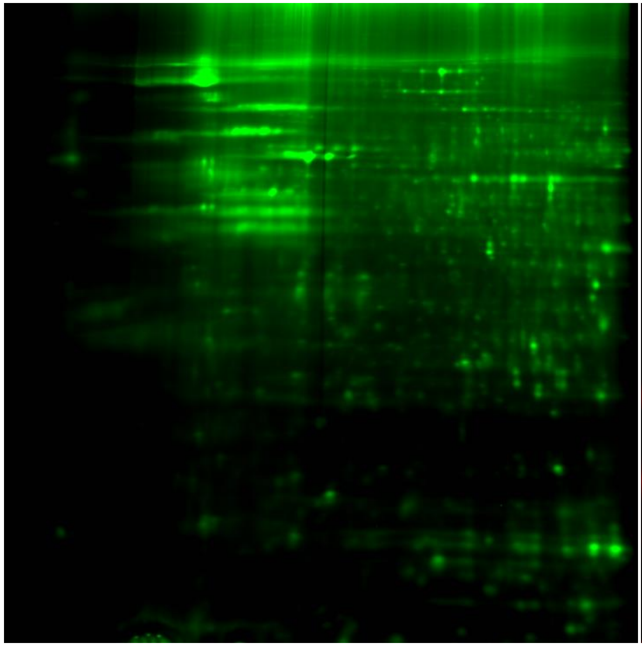

Tumor

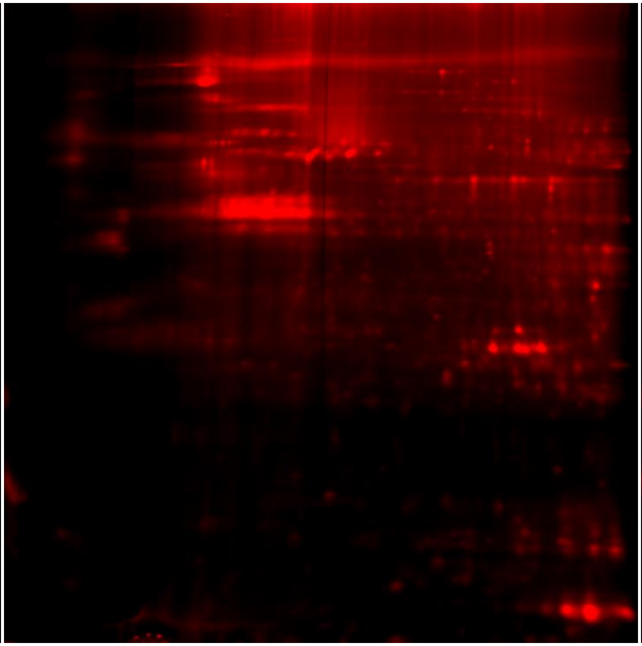

Cell / Tumor

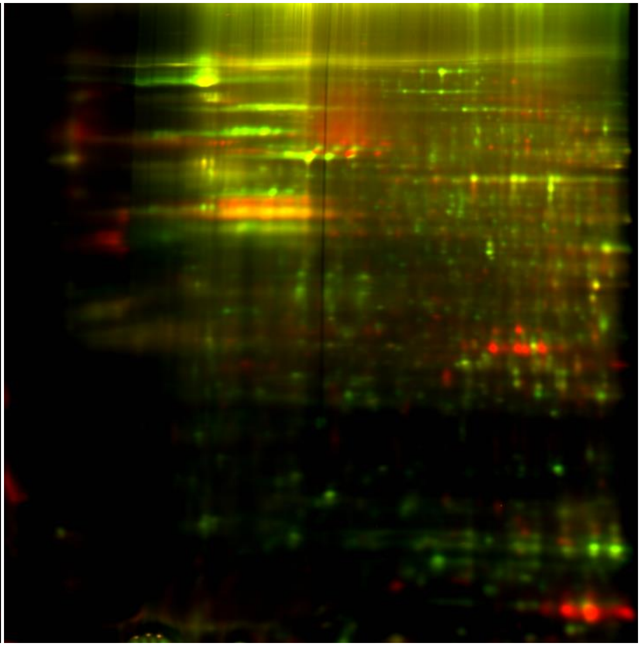

Gel-3

Cell / Tumor

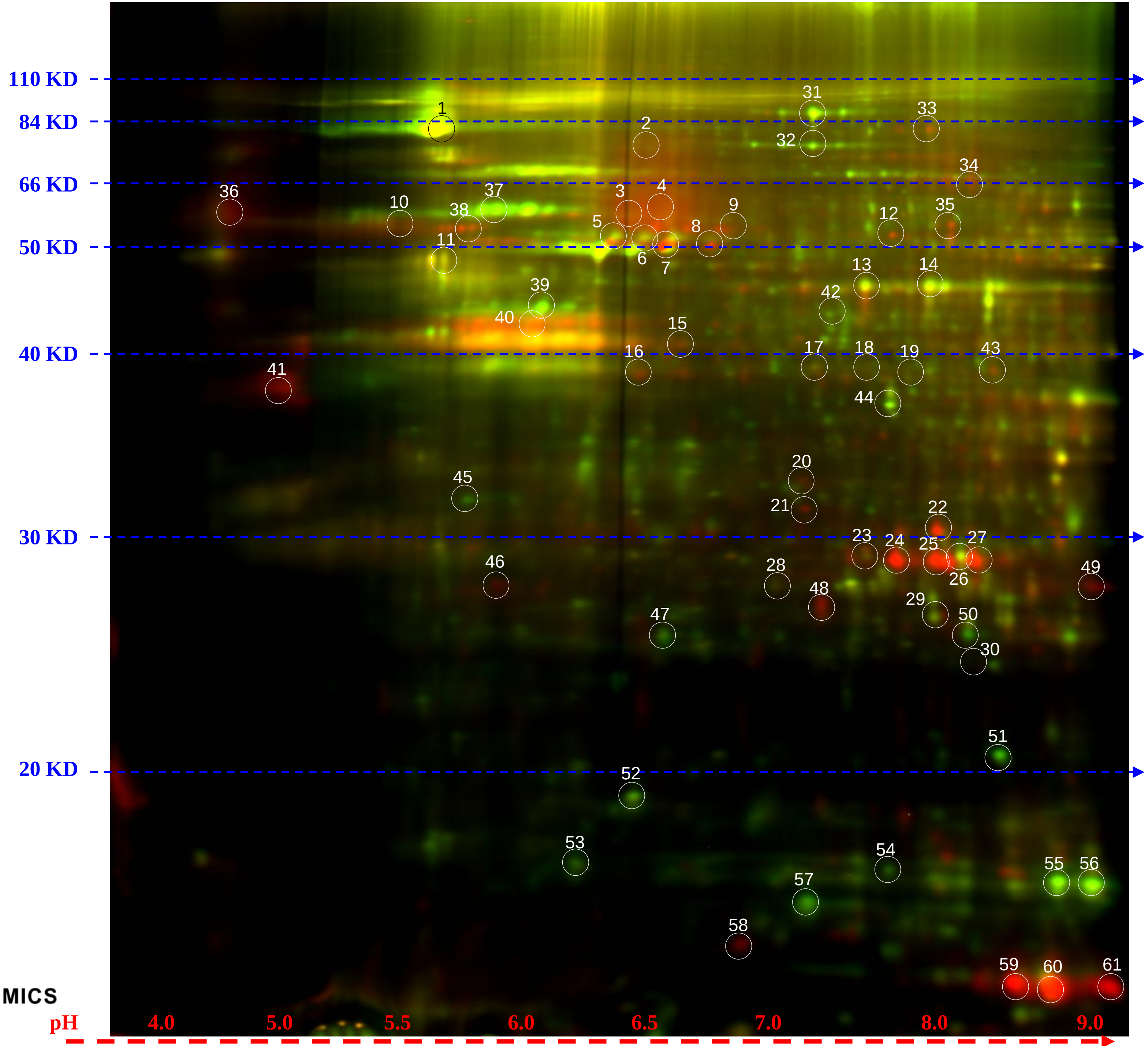

Applied Biomics
